# The Phenoxyalkyltriazine Antagonists for 5-HT_6_ Receptor with Promising Procognitive and Pharmacokinetic Properties In Vivo in Search for a Novel Therapeutic Approach to Dementia Diseases

**DOI:** 10.3390/ijms221910773

**Published:** 2021-10-05

**Authors:** Sylwia Sudoł, Agnieszka Cios, Magdalena Jastrzębska-Więsek, Ewelina Honkisz-Orzechowska, Barbara Mordyl, Natalia Wilczyńska-Zawal, Grzegorz Satała, Katarzyna Kucwaj-Brysz, Anna Partyka, Gniewomir Latacz, Agnieszka Olejarz-Maciej, Anna Wesołowska, Jadwiga Handzlik

**Affiliations:** 1 Faculty of Pharmacy, Medical College in Krakow, Jagiellonian University, PL 30-688 Kraków, Poland; s.sudol@doctoral.uj.edu.pl (S.S.); ewelina.honkisz@uj.edu.pl (E.H.-O.); katarzyna.kucwaj@uj.edu.pl (K.K.-B.); glatacz@cm-uj.krakow.pl (G.L.); agnieszka.olejarz@uj.edu.pl (A.O.-M.); 2 Department of Clinical Pharmacy, Medical College, Jagiellonian University, PL 30-688 Kraków, Poland; agnieszka.cios@uj.edu.pl (A.C.); m.jastrzebska-wiesek@uj.edu.pl (M.J.-W.); natalia.wilczynska@uj.edu.pl (N.W.-Z.); annairena.partyka@uj.edu.pl (A.P.); a.wesolowska@uj.edu.pl (A.W.); 3 Department of Pharmacobiology, Medical College, Jagiellonian University, PL 30-688 Kraków, Poland; barbara.mordyl@uj.edu.pl; 4 Department of Medicinal Chemistry, Maj Institute of Pharmacology Polish Academy of Sciences, PL 31-343 Kraków, Poland; satala@if-pan.krakow.pl

**Keywords:** 5-HT_6_ ligands, 1,3,5-triazine, ADME-Tox parameters, procognitive effects, Alzheimer’s disease

## Abstract

Among the serotonin receptors, one of the most recently discovered 5-HT_6_ subtype is an important protein target and its ligands may play a key role in the innovative treatment of cognitive disorders. However, none of its selective ligands have reached the pharmaceutical market yet. Recently, a new chemical class of potent 5-HT_6_ receptor agents, the 1,3,5-triazine-piperazine derivatives, has been synthesized. Three members, the *ortho* and *meta* dichloro- (**1**,**2**) and the unsubstituted phenyl (**3**) derivatives, proved to be of special interest due to their high affinities (**1**,**2**) and selectivity (**3**) toward 5-HT_6_ receptor. Thus, a broader pharmacological profile for **1–3**, including comprehensive screening of the receptor selectivity and drug-like parameters in vitro as well as both, pharmacokinetic and pharmacodynamic properties in vivo, have been investigated within this study. A comprehensive analysis of the obtained results indicated significant procognitive-like activity together with beneficial drug-likeness in vitro and pharmacokinetics in vivo profiles for both, (*RS*)-4-[1-(2,3-dichlorophenoxy)propyl]-6-(4-methylpiperazin-1-yl)-1,3,5-triazin-2-amine (**2**) and (*RS*)-4-(4-methylpiperazin-1-yl)-6-(1-phenoxypropyl)-1,3,5-triazin-2-amine (**3**), but insensibly predominant for compound **2**. Nevertheless, both compounds (**2** and **3**) seem to be good Central Nervous System drug candidates in search for novel therapeutic approach to dementia diseases, based on the 5-HT_6_ receptor target.

## 1. Introduction

**Figure 1:**
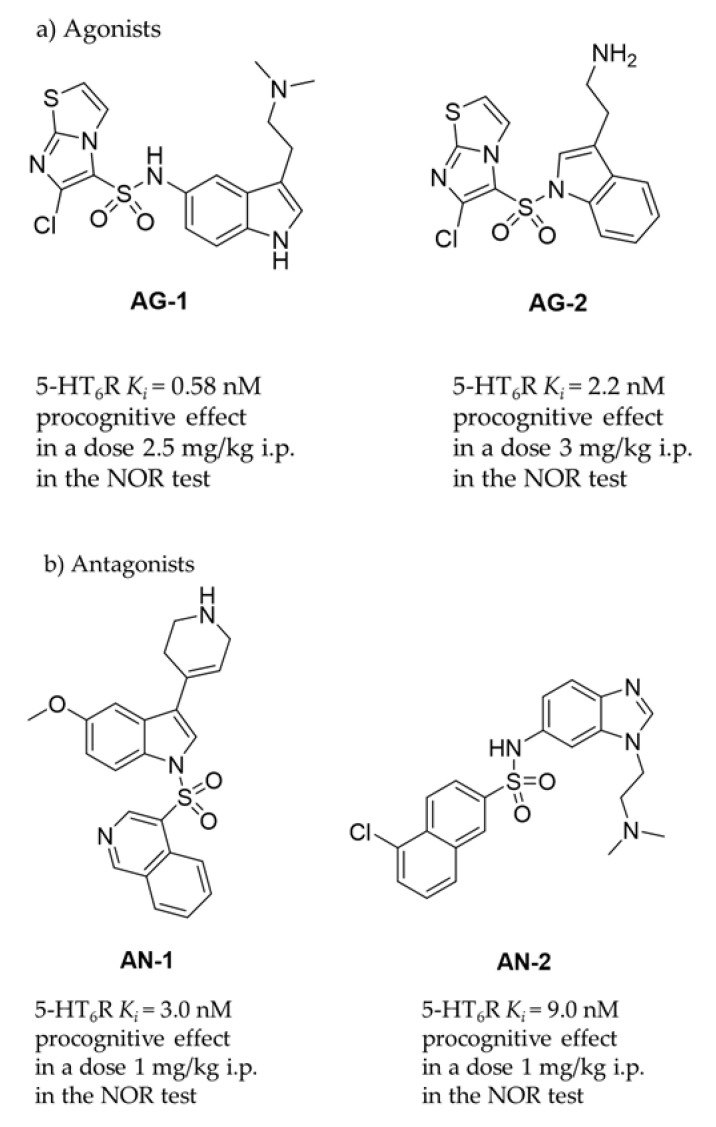

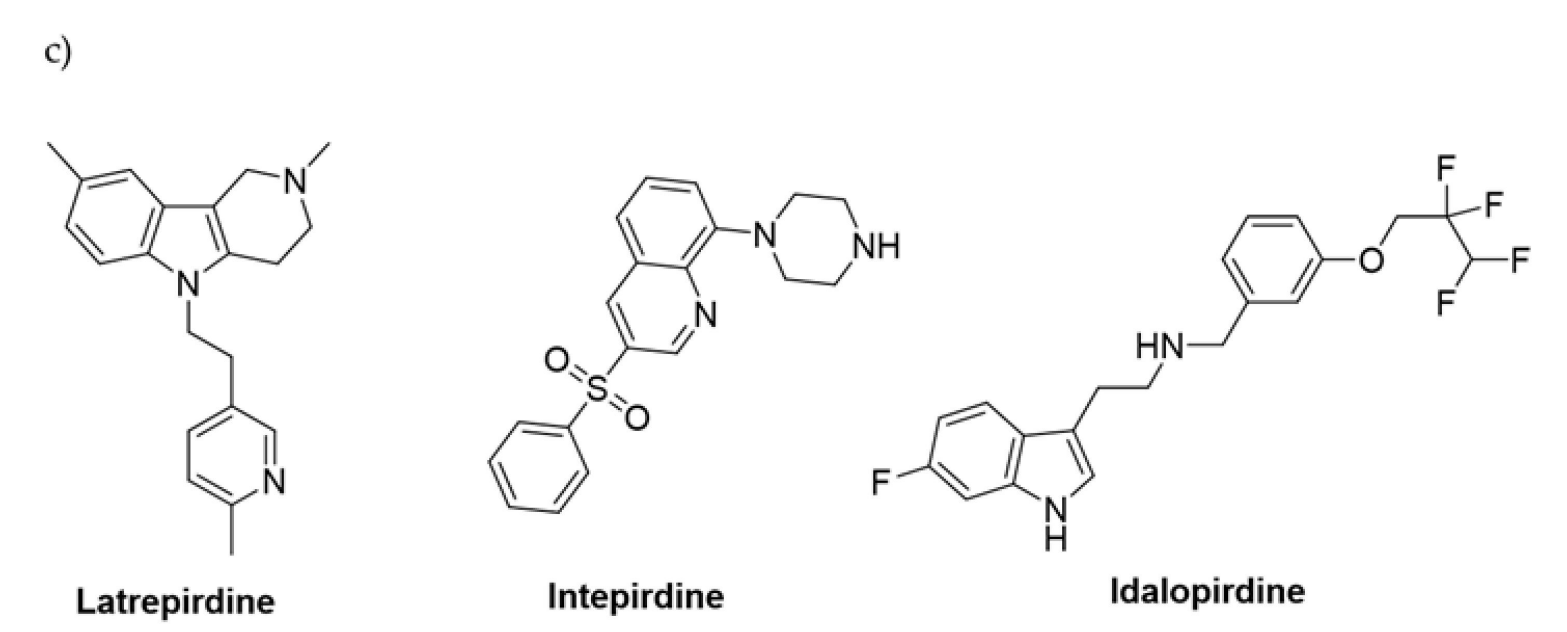
The 5-HT_6_R agents with procognitive properties. Compounds active in NOR test in primary pharmacological screening: (**a**) agonists **AG-1** [10] and **AG-2** [11]; (**b**) most active antagonists **AN-1** [12], **AN-2** [13]; (**c**) compounds that have reached clinical trials and failed in Phase III.

Central nervous system (CNS) disorders, such as dementia and Alzheimer’s disease (AD), occupy a significant place among civilization diseases. They have become a growing problem in our society due to the ever-increasing frequency of occurrence and the lack of access to effective treatment therapies. A neurodegenerative disease with a complex etiology such as AD is the most common memory dysfunction that particularly affects the elderly. These disorders are caused by the malfunctioning of proteins responsible for signal transmission in the body, mainly within the brain. Protein targets, including serotonin receptors, may be involved in the pathophysiological processes leading to AD disease, and therefore ligands of these receptors could be useful in the potential therapy. Among them, the 5-HT_6_ receptor (5-HT_6_R), one of the latest discovered in this group (1993 in rats, 1995 in human) [1,2], is of great interest for medicinal chemists due to its role in molecular mechanisms associated with civilization diseases, i.e., depression, dementia, schizophrenia and obesity [3,4,5,6,7,8]. To date, a number of chemical compounds with 5-HT_6_R affinities, including those displaying procognitive action in animal models, has been described [9]. As the models are concerned, the novel object recognition (NOR) test is one of the most common behavioral approach determining primary procognitive properties of compounds in vivo in rats. Lines of evidence indicates that the most active 5-HT_6_R agents are able to reverse memory disturbances in NOR test at the dose as low as 2.5 mg/kg i.p. in the case of agonists (**AG-1**, **AG-2**, Figure 1a) [10,11] or 1 mg/kg i.p. in the case of antagonists (**AN-1**, **AN-2**, Figure 1b) [12,13].

Among a number of chemical compounds acting on 5-HT_6_R, a vast majority bases on either sulfone or indole scaffold (Figure 1) [14], and none has yet been approved as a medicine. Unfortunately, most of the 5-HT_6_R agents that reached clinical trials (Figure 1c) failed mainly due to an insufficient effectiveness in patients despite very promising properties in preclinical studies [9]. Currently available drugs for AD are primarily used only to reduce symptoms or control behaviour, but not cure AD. Majority of them target neurotransmitter systems that include cholinergic, non-cholinergic, glutamatergic and their combinations. Most approved AD therapies have been applied more than 10 years ago, and moreover since 2000 and the memantine approval, no further compound has been authorized for AD connected cognitive deficits. Therefore, there is still urgent need of ongoing search for new compounds and treatments which will demonstrate grater efficacy in cognitive dysfunction connected with AD and related neurodegenerative diseases of aging. Moreover, the last literature dates indicate that it is important not to forego efforts to improve also the symptoms in patients at various stages of AD: from Mild Cognitive Impairment (MCI) to late-stage AD via mechanisms that not necessarily are directly involved into the pathological foundations of this disease [15]. Hence in our studies we assess also potential antidepressant- and anxiolytic-like activity of new compounds with procognitive potential.

Previously reported preclinical as well as clinical studies indicate that serotonergic system modulates memory processes [16]. Moreover 5-HT_6_ receptors are widely pursued target for various central nervous system disorders (i.e., depression, anxiety, learning and memory). The 5-HT_6_ receptors location is restricted to the specific brain regions (nucleus accumbens, striatum, hippocampus and olfactory tubercle [17,18]) on the non-serotonergic neurons—the brain areas involved in learning and memory processes and mood. Moreover, the preclinical studies support that 5-HT_6_ receptors ligands could improve memory and cognition and help to alleviate behavioral symptoms (i.e., mood disorders) of the AD in mice rats and primates in a board spectrum of behavioral paradigms: spatial learning and memory, associative learning and memory, autoshaping, attentional set-shifting, novel object discrimination or social recognition and these ligands are effective in both young and old animals [15].

The low chemical diversity limited to indole- and sulfone-containing structures (Figure 1) seems to be a reason of the clinical failures from a molecular point of view. The narrow chemical space may be translated into the triggering of similar signal transduction pathways involving the 5-HT_6_R target as well as into interactions with corresponding off-targets. Consequently, this can result in unsatisfactory pharmacodynamic and pharmacokinetic properties of the most advanced 5-HT_6_R agents in patient and the divergency observed in in vitro and in vivo models vs. clinical studies. While a difference in drug efficacy between clinical and preclinical trials is not any unique pharmaceutical problem, the question of atypical structure-dependent pharmacological effects is particularly underlined for 5-H_6_R ligands due to so called “the agonist/antagonist paradox” [19], i.e., the similar and profitable pharmacological effects demonstrated by both agonists and antagonists in a vast number of behavioral in vivo studies, including models of depression, anxiety and dementia in animals. Lines of evidence postulate several hypotheses to explain this paradox, e.g., the hypotheses based on either the functional- or regional selectivity of appropriate 5-HT_6_R ligands, as well as those based on additional ligand-specific actions on different off-targets resulting in a series of opposing cellular responses giving a similar joint effect [4]. All of the hypotheses, however, boil down to one common conclusion that the pharmacological effect is closely related to the chemical properties of a given 5-H_6_R ligand. Hence, the search for ligands in the chemical area extended beyond the predominant indole-sulfone space gives a hope to find the 5-HT_6_R agent that will successfully pass clinical exams to find application in innovative CNS therapies.

**Figure 2:**
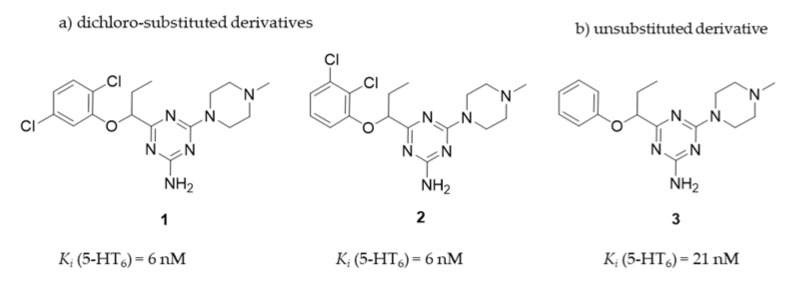
The chemical structures and 5-HT_6_R affinity of 1,3,5-triazine derivatives: (**a**) dichloro-substituted derivatives (**1, 2**); (**b**) unsubstituted derivative (**3**) [21].

In this context, we initiated to explore the group of 1,3,5-triazine-methylpiperazines as a new chemical space that meets criteria of the pharmacophore model for 5-HT_6_R antagonists developed by Lopez-Rodriguez et al. [20] but contains neither sulfone group nor indole-like moiety. Results of those studies allowed to find a series of very potent derivatives with the high affinity (*K_i_* < 100 nM) for 5-HT_6_R [21]. From the obtained compounds, the highly potent 5-HT_6_R antagonist, compound **1**, was selected as a lead structure and preliminary evaluated in ADMET studies in vitro and a rat behavioral model in vivo. Results have shown that **1** exhibited excellent permeability in the parallel artificial membrane permeability assay (PAMPA) as well as it gave procognitive properties tested at the dose of 3 mg/kg i.p. in NOR test in rats. However, its “drug-like” profile was not very satisfactory due to a rather weak metabolic stability and some hepatotoxic effects caused at higher concentrations. Apart from compound **1**, two other members of the series [10] took our attention, i.e., compound **2**, the 2,3-chlorosubstituted isomer of **1**, showing the same affinity for the 5-HT_6_R (*K_i_* = 6 nM), and the phenyl-unsubstituted compound **3** with slightly weaker affinity (*K_i_* = 21 nM) but the high selectivity for 5-HT_6_R with respect to other off-targets (Figure 2). Both compounds, **2** and **3**, were selected for further consideration within this study.

Taking into account the previous results in the early stages of the primary pharmacological screening for all the compounds, and the basic assessment of “drug-likeness” for **1** [21], we decided to extend the biological tests to judge if the pharmacodynamic (procognitive-like) potency, pharmacokinetic and a general “drug-like” profile, give therapeutic perspectives for compounds **1–3** in potential treatment of dementia diseases. Thus, this work investigated a broader receptor binding profile for **2** and **3** in comparison to that of **1**, including their functional intrinsic activity towards 5-HT_6_R and 5-HT_2A_R, and a comprehensive in vitro evaluation of their ADMET properties. ADME in vivo studies to determine pharmacokinetic parameters and tissue-distribution of compounds **2** and **3**, as well as behavioral studies in rats to assess the potential procognitive, antidepressant- and anxiolytic-like effects of **2** and **3** with respect to **1** were carried out. Finally, a comprehensive discussion was performed to evaluate how strongly molecular differences affect the efficacy of these triazine 5-HT_6_R antagonists as potential compounds which could improve memory disturbances and help alleviate behavioral symptoms of AD in the future.

## 2. Results

### 2.1. Pharmacology In Vitro

#### 2.1.1. Radioligand Binding Assay

Previously, the 5-HT_6_R ligands **1–3** were tested in radioligand binding assays (RBA) on their affinity and selectivity towards the human 5-HT_6_R with respect to off-targets 5-HT_2A_R, 5-HT_7_R and the dopaminergic receptor D_2_ [10]. In order to extend knowledge about their receptor profile, an assay for 5-HT_1A_R has been performed in these studies as well. The combined results indicated that both highly active 5-HT_6_R ligands **1** and **2** (*K_i_* = 6 nM) displayed also submicromolar affinities to the off-targets, i.e., 5-HT_1A_R (**2**), 5-HT_2A_R (**1**, **2**) and 5-HT_7_R (**1**, **2**). Although all the examined derivatives (**1–3**) had a distinct selectivity over the off-targets, a risk of an action on the off-targets can be disregarded only in the case of compound **3** due to the micromolar/submillimolar binding effects vs. the much stronger nanomolar binding (*K_i_* = 21 nM) for the 5-HT_6_R (Table 1).

In the light of the current trends promoting “poly-pharmacology”, additional interactions with the considered GPCRs may even be beneficial for potential therapy, strengthening antidepressant (5-HT_1A_, 5-HT_7_) and mood enhancing (D_2_) or procognitive effects (5-HT_7_) [9]. An exception is the 5-HT_2A_, which carries a risk of neurotoxic undesirable effects in the case of agonistic action. Therefore, it was a very important aspect to determine intrinsic activity of the compounds (**1–3**) with special attention to the action of **1** and **2** on the 5-HT_2A_R.

#### 2.1.2. Functional Assays

Previous functional assays, performed only for **1** towards 5-HT_6_R [21], have been extended to **2** and **3**, including the evaluation of intrinsic activity for 5-HT_6_R (**2**, **3**) and 5-HT_2A_R (**1**, **2**) with respect to RBA results. During the experiments, the level of cAMP was measured. Results are shown in Table 2. Compound **1** revealed the strongest antagonistic action towards 5-HT_6_R (*pK_b_* = 10.57) [21] and the partly agonistic mechanism towards 5-HT_2A_R. Comparing to **1**, compounds **2** and **3** displayed slightly weaker antagonistic action on 5-HT_6_R in the order consistent with their results in RBA. It is worth emphasizing that in the case of both, **2** (*pK_b_* = 8.19) and **3** (*pK_b_* = 7.67), the antagonistic effect was comparable with that of reference SB258585. Finally, it is of great importance that compound **2**, the most potent 5-HT_2A_R agent in RBA, demonstrated antagonistic properties even stronger for 5-HT_2A_R (*pK_b_* = 8.61) than those found for 5-HT_6_R (*pK_b_* = 8.19).

### 2.2. ADMET Assays In Vitro

Based on the preliminary data for compound **1** in our previous work [21], in vitro “drug-likeness” studies have been performed also for compounds **2** and **3** including aspects of permeability, metabolic stability and safety.

#### 2.2.1. Permeability

The penetration through biological membranes is the crucial aspect determining in vivo activity of CNS targeting compounds. Thus, compounds **1**–**3** were tested in PAMPA for their ability to the passive transport.

The results of PAMPA were declared as permeability coefficient *Pe* and compared with the highly permeable agent (caffeine, CFN, Table 3).

The examined compounds **2** and **3** demonstrated an excellent permeability, with *Pe* values corresponding (**3**) or higher (**1**, **2**) than that of high-permeable CFN (*Pe* = 15.1 × 10^−6^ cm/s), and much higher than the breakpoint for permeable compounds according to manufacturer’s guideline (*Pe* ≥ 1.5 × 10^−6^ cm/s) [22]. The Pe measured for **2** (*Pe* = 20.5 × 10^−6^ cm/s) was even higher than that estimated in the previous studies for **1**. The results prove that the susceptibility of the triazine derivatives (**1–3**) for the passive transport through biological membranes depends on either presence or positions of the chlorine substituents. Thus, 2,3-dichloro (**2**) substitution seems to be more profitable than the 2,5-dichloro (**1**) one, while both (**1,2**) give an increase of permeability in comparison to the phenyl-unsubstituted derivative (**3**).

#### 2.2.2. Caco-2 Permeability Assay

The Caco-2 permeability assay is considered to be representative of human absorption in vivo as it provides a good prediction for compounds that display active uptake, efflux or pass through the membrane via the paracellular route. As shown in Table 4 compound **2** has an excellent permeability in Caco-2 conditions with *Papp* value 50.3 ± 6.22 for A-B direction, while *Papp* value 21.1 ± 2.14 for B-A does not suggest an efflux mechanism. Compound **1** has also excellent permeability (*Papp* value 14.2 ± 0.36 for A-B) close to caffeine and permeates only by passive diffusion. Interestingly, compound **3** exhibits moderate permeability with the *Papp* value 3.16 ± 1.35 for A-B and has an efflux ration greater than **2** (3.20) which suggests that the compound may be a subject to active efflux.

#### 2.2.3. Metabolic Stability In Vitro

**Figure 3:**
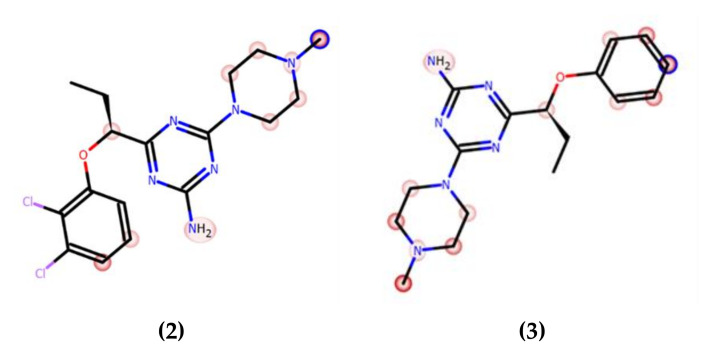
In silico prediction of the sites of metabolism by MetaSite 8.0.1 for **2** and **3**. Blue circle marked on the functional group structures indicates the highest biotransformation probability. The fading red color shows the decreasing of the metabolism probability.

The correlation between the microsomal metabolism of a compound in vitro in the liver and its downstream pathway in vivo is a crucial parameter at the early stage of drug discovery in order to understand pharmacokinetic properties. Thus, the metabolic stability was investigated in vitro by using rat liver microsomes (RLMs) and computer-aided with MetaSite 8.0.1 software. The most probable structures of metabolites were estimated. In silico predicted sites of biotransformation for **2** and **3** were specified for *N*-methylpiperazine moiety and for **2** *ortho* position of the chlorine di-substituted aromatic ring (Figure 3, Table 5).

In the previous studies, after the incubation with RLMs for 120 min the formation of six metabolites was observed, and more than 90% of **1** was biotransformed [21]. In silico predicted sites of metabolism for **1** were specified for *N*-methylpiperazine moiety and *para* position of the 2,5-dichloro-substituted aromatic ring, including the hydroxylation at the phenyl ring, demethylation and hydroxylation of piperazine. To assume, the previously obtained results illustrated low metabolic stability for **1**.

**Figure 4:**
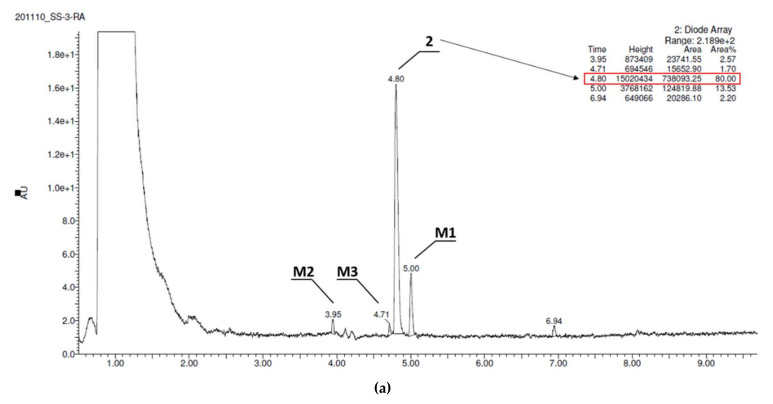

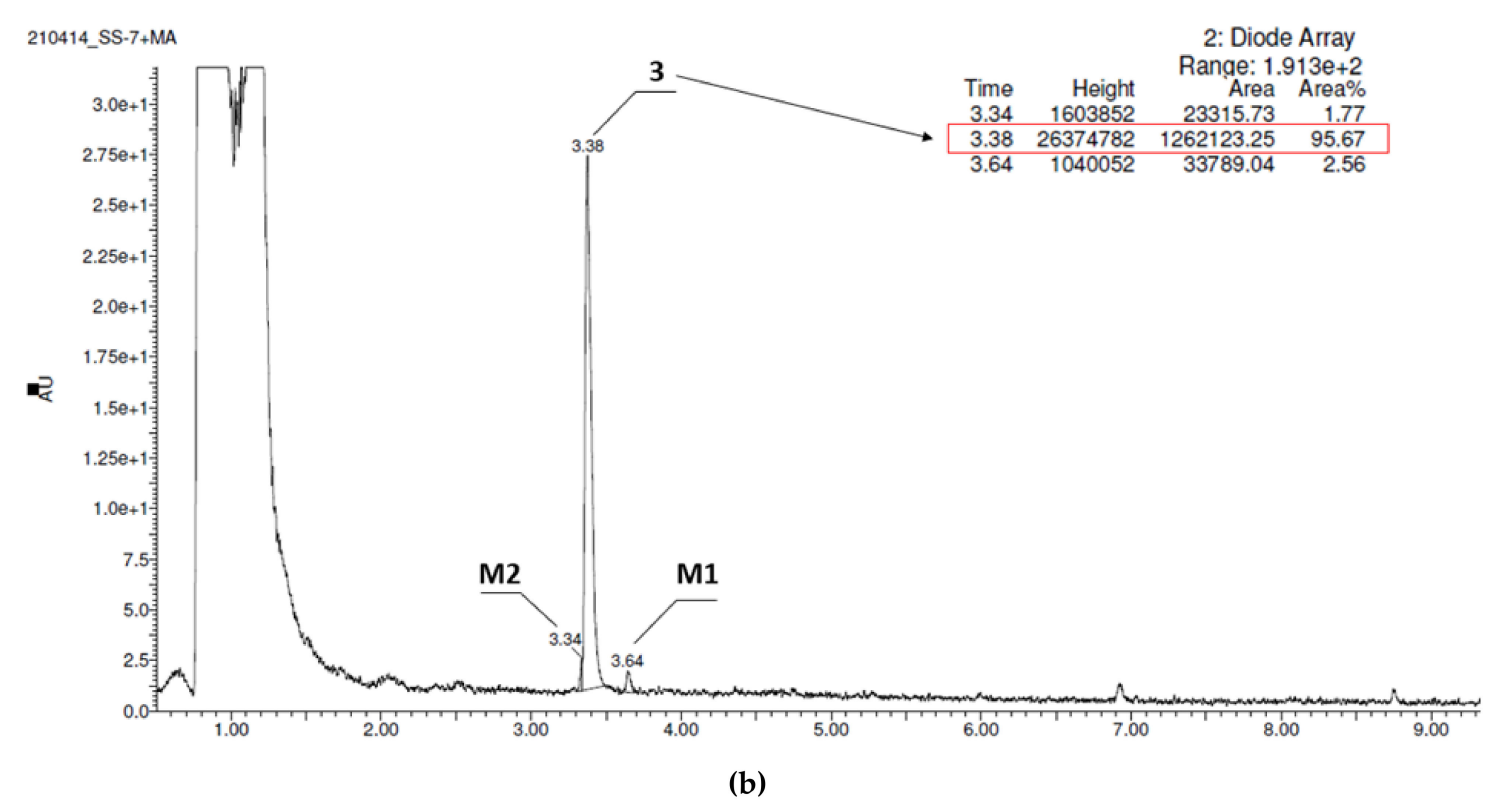
UPLC chromatograms of the reaction mixtures after 120 min incubation of compounds with RLMs (**a**) **2**, (**b**) **3**. Estimated stability ratio (**2** vs. **3**)~0.84.

In contrast, compound **3**, the derivative without chlorine substituents, was found to be metabolically stable (95,67% of **3** remained in the reaction mixture, Figure 4a), whereas **2**, the 2,3-dichloro structural isomer of **1**, also improved the resistance for metabolizing enzymes and displayed moderate stability (20% of the parent structure was biotransformed, Figure 4b). Furthermore, the number of metabolites formed was as low as three in the case of **2**, and two in the case of **3** (Table 5). The MS spectra analysis and in silico data allowed to establish the most probable metabolic pathways of the two tested 5-HT_6_R ligands (Figures S1B–S2C in Supplementary Materials, Table S4).

#### 2.2.4. Safety

Safety studies of compounds **1–3** were performed in vitro with recombinant cytochrome P450 (CYP) isoform, *hepatoma* HepG2 cell line and the bacterial strain *Salmonella* Typhimurium TA100. The growth conditions and assay protocols were followed as previously described [23]. Additionally, neurotoxic effects in neuroblastoma SHSY-5Y were examined.

##### The Effect on Cytochrome P450

**Figure 5:**
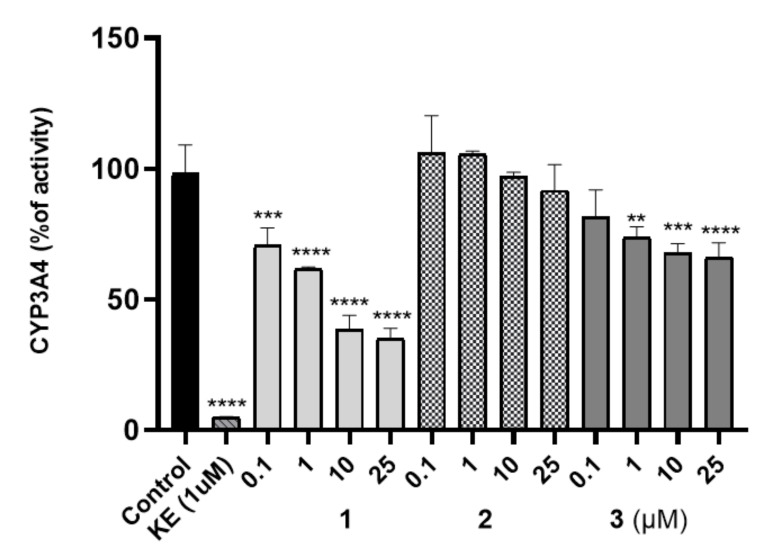
Influence of **1**–**3** on CYP3A4 activity, KE—ketoconazole. Statistical significance was evaluated by one-way ANOVA, followed by Bonferroni’s comparison test (** *p* < 0.01, *** *p* < 0.001, **** *p* < 0.0001) using GraphPad Prism 8.0.1.

The potential risk of drug-drug interactions (DDI) was tested by luminescence-based CYP3A4 P450-Glo™ assays (Promega^®^). This isoform is involved in the metabolism of relatively half of all available medicaments, and evaluation of the effects on activity is important. The influence of **1–3** on the activity of isoform CYP3A4 was compared with the reference inhibitor ketoconazole (KE, Figure 5) used as a positive control. Compound **2** did not display an inhibition against CYP3A4 including all tested concentrations (0.1 to 25 μM) in comparison to the control reaction and the respective reference inhibitor. CYP3A4 activity was decreased by **3** at concentrations of 1 to 25 μM. However, the CYP3A4 was inhibited by 10 μM of **1** up to 44% of activity whereas by 10 μM of **3** only to 64%. Moreover, it is worth pointing out that the inhibitory activities of the tested compound against CYP3A4 were lower than the activities of ketoconazole.

##### Hepatotoxicity

**Figure 6:**
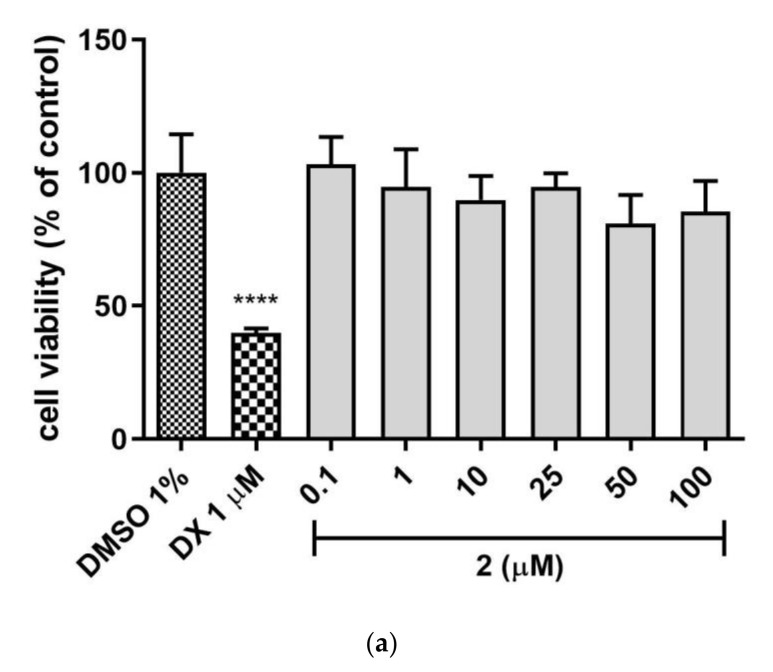

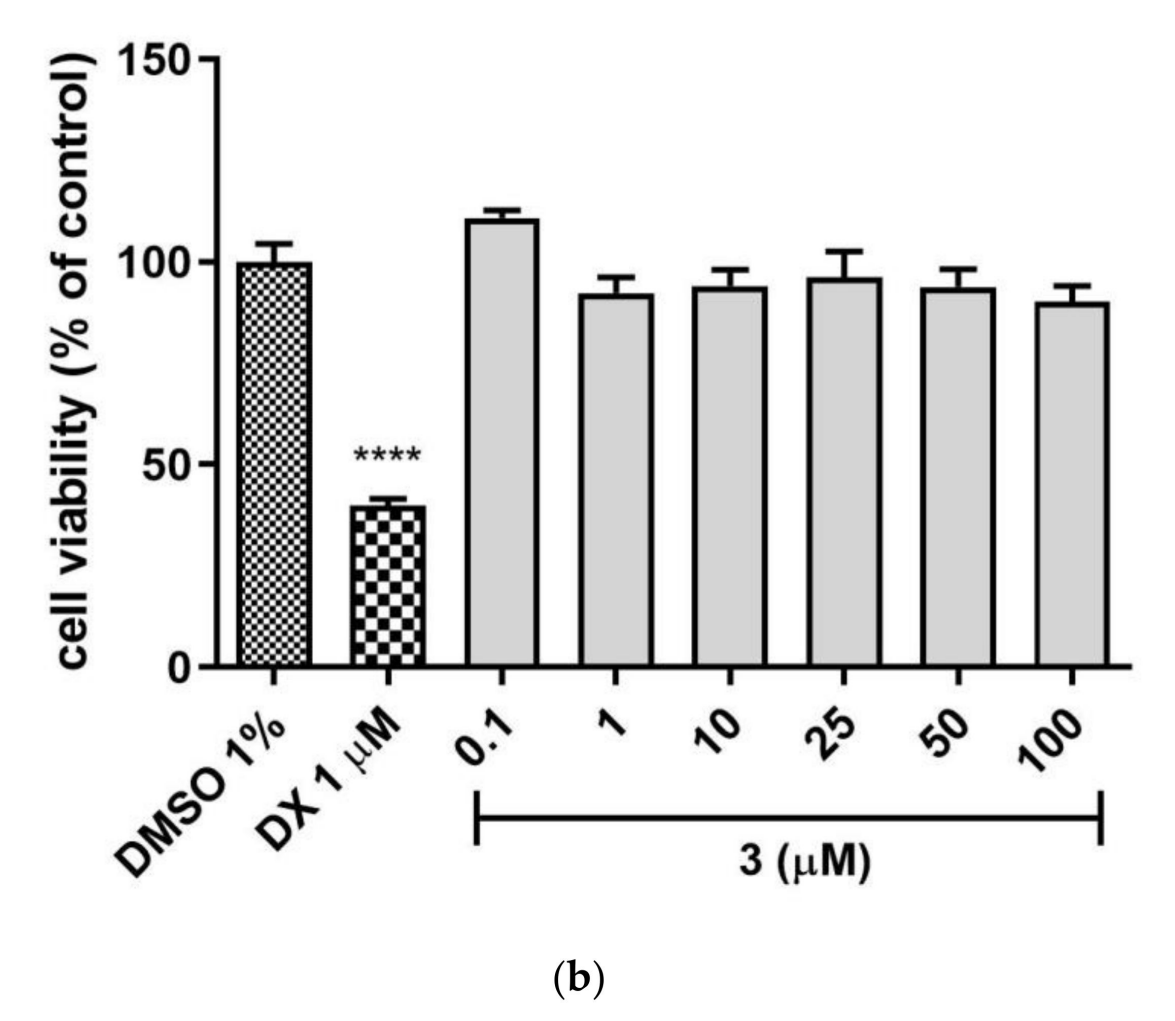
The effect of tested compounds (**a**) 2, (**b**) 3 and reference: doxorubicin (DX, 1 µM) on hepatoma HepG2 cell line viability. 1% DMSO in cell growth media was used as a negative control. GraphPad Prism 8.0.1 was used to calculate the statistical significances by one-way ANOVA, followed by Bonferroni’s comparison test (**** *p* < 0.0001).

The safety profile of the **2** and **3** was estimated in the hepatotoxicity assay in vitro using hepatoma HepG2 cell line, according to the protocol reported earlier [24,25,26,27,28]. Both tested compounds **2** and **3** demonstrated no hepatotoxicity effect when compared to the used reference toxin (doxorubicin, DX, Figure 6a,b), while **1** displayed a weak hepatotoxic effect at 50 µM concentration [21]. The statistically significant decrease in cells viability was not observed at all tested concentrations of **2** and **3** (0.1–100 µM). Hence, both compounds displayed a satisfied in vitro safety.

##### Mutagenicity

The potential mutagenicity of **1–3** was appraised by Ames microplate fluctuation protocol (MPF). The used *Salmonella* Typhimurium T100 strain permits the detection of base-pair substitution. At first, the medium control baseline (MCB) was calculated, which means the number of revertants observed in the control (growth medium + 1% DMSO). Following the manufacturer protocol [23], the 2-fold increase over the MCB is considered as the mutagen alert. It has been not attainable in the case of examined compounds **1–3**, based on the number of revertants observed in the presence of the tested ligands. Therefore, compounds **1–3** did not display any mutagenic effects in vitro (Table 6).

##### Neurotoxicity

Neurodegenerative diseases are characterized by several complex molecular processes, one of which is oxidative stress. Therefore, the compounds **1–3** were evaluated for their protective activity in neuroblastoma SH-SY5Y cells. SH-SY5Y cells are relevant in vitro human model to detect early neurotoxicity of a drug candidate. 

**Figure 7:**
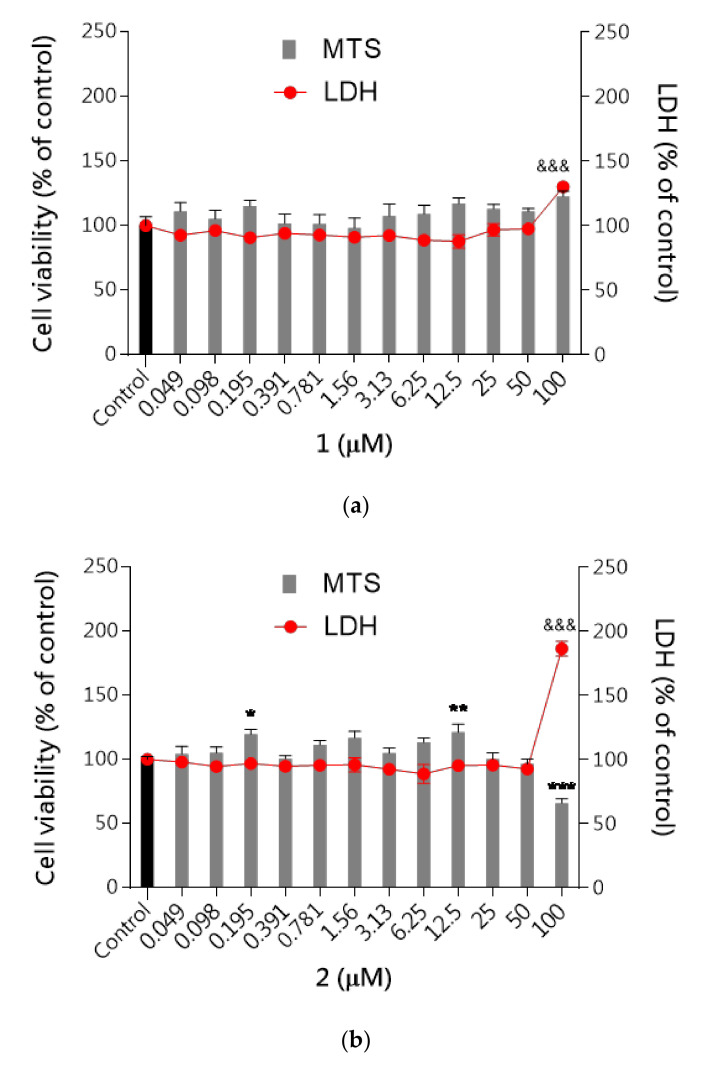

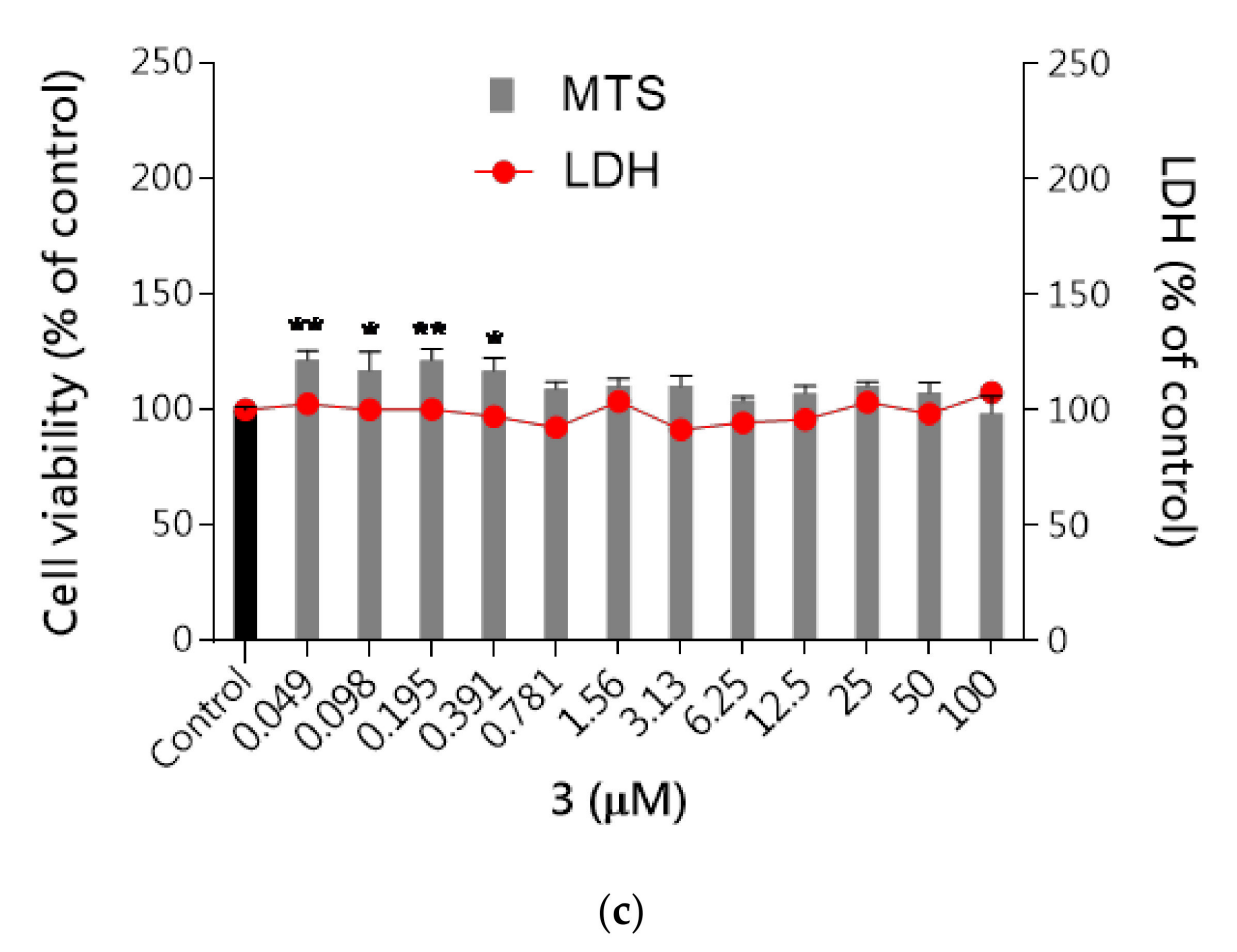
The effect of tested compounds **1–3** on neuroblastoma cell viability: (**a**) **1**, (**b**) **2**, (**c**) **3**. SH-SY5Y cells were treated with compound **1**, **2** or **3** over a wide range of concentrations (0.049–100 μM) for 48 h. Cell viability was measured by MTS assay. Each point (mean ± SEM of two independent experiments, each of which consisted of eight replicates per treatment group) represents absorbance units and is expressed as a percentage of control compared to 0.1% DMSO control cells (set as 100%). Statistical analysis by one-way ANOVA (GraphPad Prism 8) showed significant differences between the groups (*p* < 0.05) and was followed by the Dunnett’s multiple comparisons test. Data indicated with *** *p* ≤ 0.001, ** *p* ≤ 0.01, * *p* ≤ 0.05, &&& *p* ≤ 0.001 reflect statistically significant differences between control and experimental groups.

First, the cytotoxicity of studied compounds was examined using the MTS assay (assessing cell metabolic stability) and LDH assay (membrane integrity) to select the safe (nontoxic) concentrations for further analysis. As shown in Figure 7a–c none of the tested compounds evoked specific neurotoxicity in the range of tested concentrations (0.049–100 μM) except the highest concentration of 100 μM for compound **2** which significantly inhibited cell proliferation up to 65% and increased the LDH leakage by 86% compared to the control cells. The metabolic activity of the cells was close to the vehicle control (DMSO 0.1% treated cells). The results revealed that none of these compounds induced significant cytotoxicity at concentrations up to 50 μM, while at the highest concentrations 100 μM LDH leakage was observed.

##### Neuroprotective Activity

**Figure 8:**
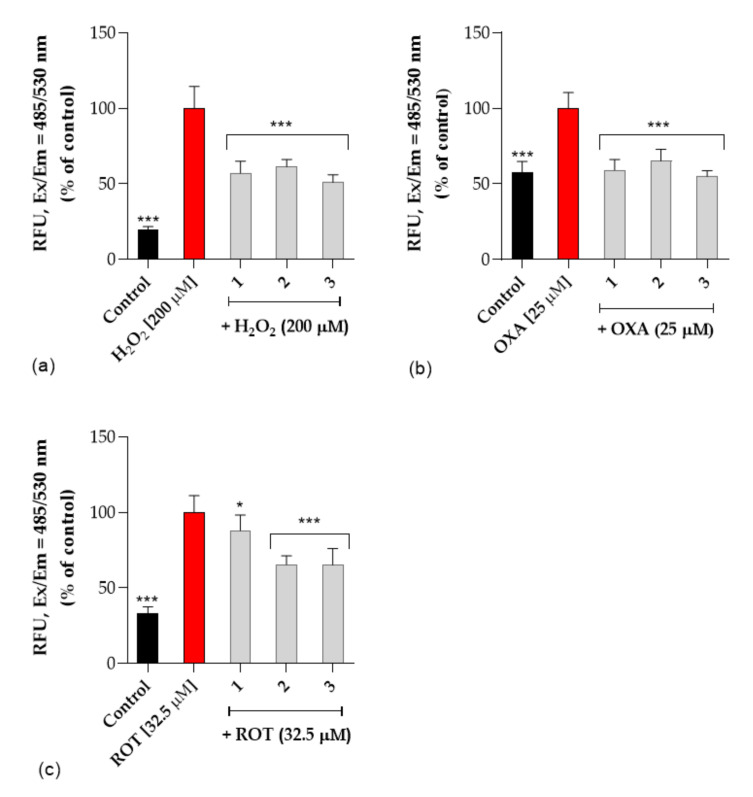
The effect of tested compounds (**1**, **2** and **3**) on (**a**) H_2_O_2_—induced ROS production, (**b**) oxaliplatin-induced ROS production (OXA, 25 μM), (**c**) rotenone-induced ROS production (ROT, 32.5 μM) in SH-SY5Y cells. Intracellular ROS production was determined by DCFH-DA assay as described in experimental procedures. Each point (mean ± SEM of two independent experiments, each of which consisted of eight replicates per treatment group) represents relative fluorescence units and is expressed as a percentage of control compared to corresponding toxin-treated cells (set as 100%). Statistical analysis by one-way ANOVA (GraphPad Prism 8) showed significant differences between the groups (*p* < 0.05) and was followed by the Dunnett’s multiple comparisons test. Data indicated with *** *p* ≤ 0.001, * *p* ≤ 0.05 reflect statistically significant differences between corresponding toxin-treated cells and experimental groups.

Next, the neuroprotective activity against three selective neurotoxins (H_2_O_2_, oxaliplatin and rotenone) was studied. Reactive oxygen species (ROS) cause oxidative stress that is thought to play an important role in neurodegenerative diseases. To verify the possible neuroprotective effect of the tested compounds against neurotoxicity induced in SH-SY5Y cells, DCFH-DA assay was performed. All tested compounds **1–3** were studied at concentrations of 25μM based on cytotoxicity determined with SH-SY5Y cells. We found that the tested compounds alone did not affect ROS production. Treatment with neurotoxins markedly increased the intracellular level of ROS in SH-SY5Y cells and pre-treatment with the tested compounds significantly prevented ROS production by 2-fold compared to the neurotoxins-treated cells (Figure 8). Compounds **1–3** exert protective properties against rotenone-induced neuronal toxicity as well as against H_2_O_2_ and oxaliplatin via reduced oxidative stress. The ROS level reduction in the cell examined by the DCFH-DA method was reduced to 50% compared to cells treated with selected neurotoxins. This observation is of particular significance considering the involvement of ROS in the development of neurodegenerative disorders.

### 2.3. Pharmacokinetics In Vivo

These studies also include the in vivo pharmacokinetic profile of compounds **2** and **3** for which tissue distribution has been determined and the concentrations are plotted and values given in the tables. The current HPLC methods were developed and validated for the determination of **2** and **3** in rat serum and tissues. This is the first report detailing the pharmacokinetic properties of compounds **2** and **3** after i.p. administration route in rats.

**Figure 9:**
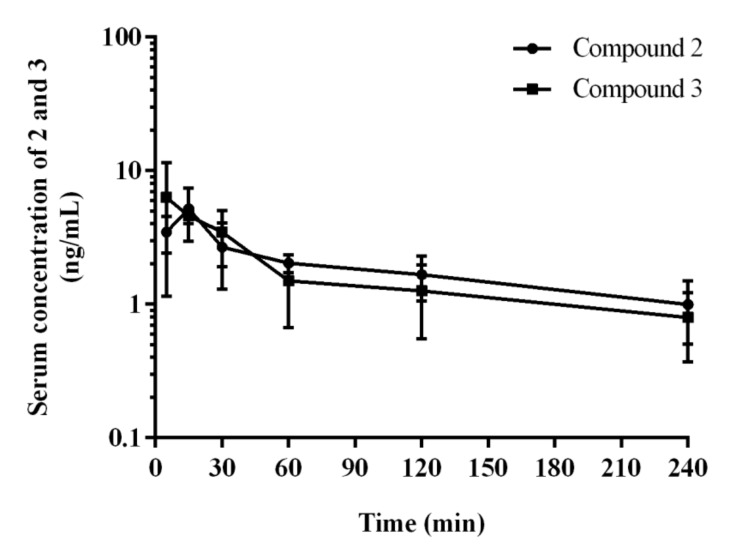
Serum concentration–time profiles of **2** and **3** in rats after at 0.3 mg/kg i.p. injection. Each point represents the mean ± SD (*n* = 4 rats/time point).

Serum concentration-time profiles of **2** and **3** after i.p. administration of a dose of 0.3 mg/kg to rats are shown in Figure 9. 

Pharmacokinetic parameters for **2** and **3** calculated by the non-compartmental approach are summarized in Table 7.

After i.p. administration, compound **2** has higher volume of distribution at the elimination phase as compared to **3** (80.9 vs. 64.8 L/kg) what might indicate its better ability to penetrate to the deep compartments. Moreover, compound **2** has longer half-life at the elimination phase (116.2 vs. 76.7 min), and lower clearance (30.9 vs. 35.2 L/h/kg) as compared to the corresponding values for **3**. 

**Figure 10:**
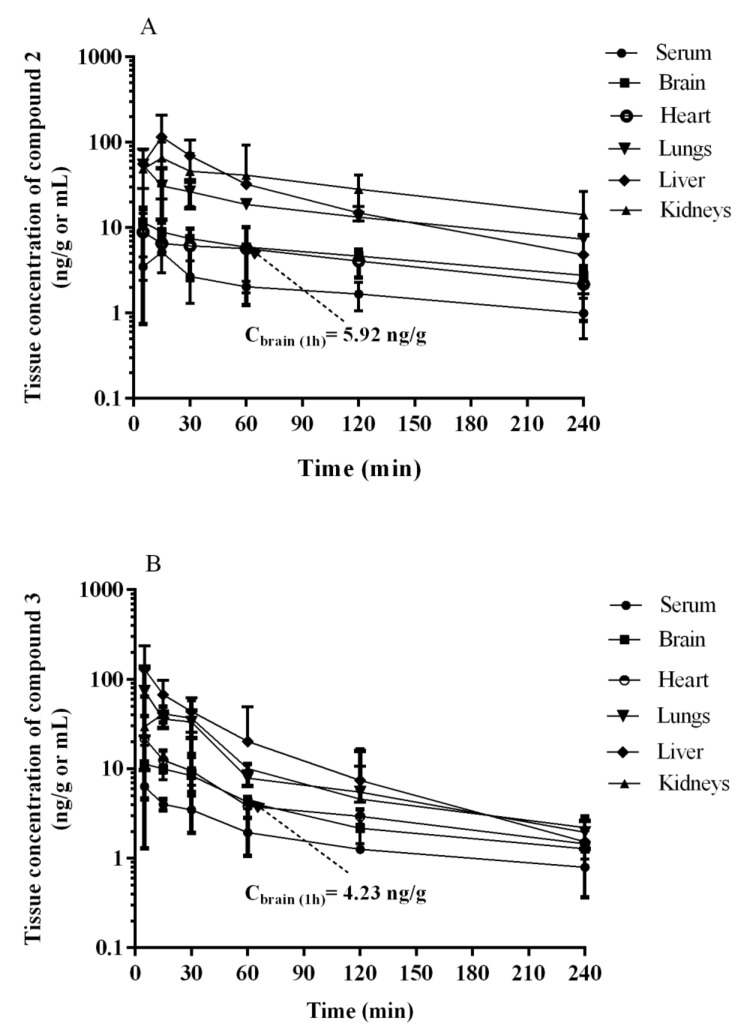
Serum and tissue concentration-time profiles of (**A**) **2** and (**B**) **3** in rats after a 0.3 mg/kg i.p. injection. Each point represents the mean ± SD, (*n* = 4 rats/time point).

The tissue concentration–time profiles of **2** and **3** after an i.p. administration of a dose of 0.3 mg/kg in rats (*n* = 4 per time point) are shown in Figure 10.

The maximum concentrations were observed at the first sampling time point, i.e., 5 min, in most tissues examined with the exception of lungs, and the value of this parameter was the highest in liver (Table 8). The examination in rats in a dosage 0.3 mg/kg after i.p. injection showed that the compound **2** (Figure 10A, Table 8) and **3** (Figure 10B, Table 8) cross the blood–brain barrier well, where the maximum concentration for compound **2** and **3** was comparable (11.63 ng/mL after 5 min vs. 11.35 ng/mL). This is in an accordance with results of both, PAMPA and metabolic stability assays in vitro. The opposing effects of better membrane permeability for compound **2** with respect to **3** and, on the other hand, the higher metabolic stability of **3**, may explain a similar distribution of both compounds (**2** and **3**) in the brain. Interestingly, the time-dependent concentration of compounds in the brain indicates a higher concentration of compound **3** compared to **2** in the first 30 min (Figure 10), then, the reversal situation can be observed. Hence, compound **2** maintained a brain concentration about twice as high as **3** until the end of the monitoring (240 min). This behavior may suggest predominant properties of **2** to longer maintain a therapeutic dose but, in general, both compounds proved beneficial blood–brain pharmacokinetic properties in this study. In contrast, it is less favorable that the hepatic concentration (C_max_) of both compounds (**2** and **3**) is relatively high. It seems to be associated with the metabolic stability of the compounds as their C_max_ ratio (0.89, Table 8) is almost equal with the in vitro metabolic stability ratio of **2** vs. **3** (0.84, Figure 4). Despite these unfavorable hepatic C_max_ in the initial minutes of the observation, the significant decrease in the concentration over the next 225 min indicates rather low risk of prolonged accumulation of both **2** and **3** in the liver. Moreover, none of the compounds showed hepatotoxic effects in vitro. In the case of **3**, there is a slight risk of DDI. However, the hepatic C_max_ is lower than the lowest concentration of **3** causing statistically significant inhibition of CYP3A4 in vitro (C_max_ = 0.395 µM vs. C_CYP3A4_ = 1 µM, Table 8 vs. Figure 5).

As the rest of pharmacokinetic parameters, the AUC_0-t_ values estimated for the studied tissues (i.e., brain, heart, lungs, liver, and kidneys) for **2** and **3** ranged from 1040.7 ng∙min/g (heart) to 7488.1 ng∙min/g (kidneys), and from 859.1 ng∙min/g (brain) to 4467.2 ng∙min/g (liver), respectively. The ratio of AUC_0-t_ for **2** and **3** in the studied tissues to that in serum (K_p_) ranged from 2.30 (heart) to 16.5 (kidneys), and from 2.03 (brain) to 10.5 (liver), with the value 2.69 and 2.03 for the brain tissue, respectively (Table 8).

Summing up, results of the assays in vivo, supported by in vitro ADMET tests, indicated a beneficial and safe pharmacokinetic profile for the investigated triazine 5-HT_6_R agents (**2**, **3**), in particular, for the 2,3-dichlorophenyl derivative (**2**). 

### 2.4. Behavioral Tests In Vivo

Following previous studies for compound **1** [17], the 1,3,5-triazine derivatives **2** and **3** were also investigated in vivo on their procognitive action in NOR test. As we mentioned above the search for potential compounds which might be useful in the AD treatment concentrates also on the substances which could improve behavioral disturbances i.e., mood disorders, connected with AD. Moreover, a lot of literature data indicate such pharmacological activity of 5-HT_6_R ligands [3,4,5,6,7,8,10,12]. Hence we decided to assess anxiolytic- and antidepressant-like properties of compounds **1, 2** and **3**, too. Thus, the compounds (**1–3**) were evaluated on their antidepressant-like activity in forced swim test (FST) and anxiolytic-like activity in the elevated plus maze (EPM) test. 

#### 2.4.1. The Impact of Memory Impairment of Investigated Compounds 

NOR test is a well-established animal test to assess episodic memory, and various 5-HT_6_R antagonists were shown to be effective in this paradigm as well as were able to reverse memory impairments induced by e.g., scopolamine, ketamine or dizocilpine (MK-801) [29]. The pharmacologically induced cognitive deficits in animals are believed to the model of the impairments seen in humans such as: a consequence of developmental intellectual disabilities, aging, or disease processes [30]. Agents that ameliorate these cognitive functions and display a characteristic pharmacological profile are called “cognition enhancers”. MK-801 is experimentally widely used NMDA receptor antagonist, and MK-801-induced cognitive impairments have been validated as a rodent model related to human cognitive deficits associated with dementia [31] and schizophrenia [32]. The MK-801-induced cognitive deficits can be antagonized by putative cognition enhancers. The literature provides ample evidence that cognition enhancers with different modes of action are able to ameliorate these deficits [29,31,33,34], and among them also antagonists as well as agonists of 5-HT_6_ receptors [35]. These data support the notion that animal models with cognitive deficits induced by MK-801 may possess some predictive validity in the search of new compounds with potential impact on the memory and learning.

**Figure 11:**
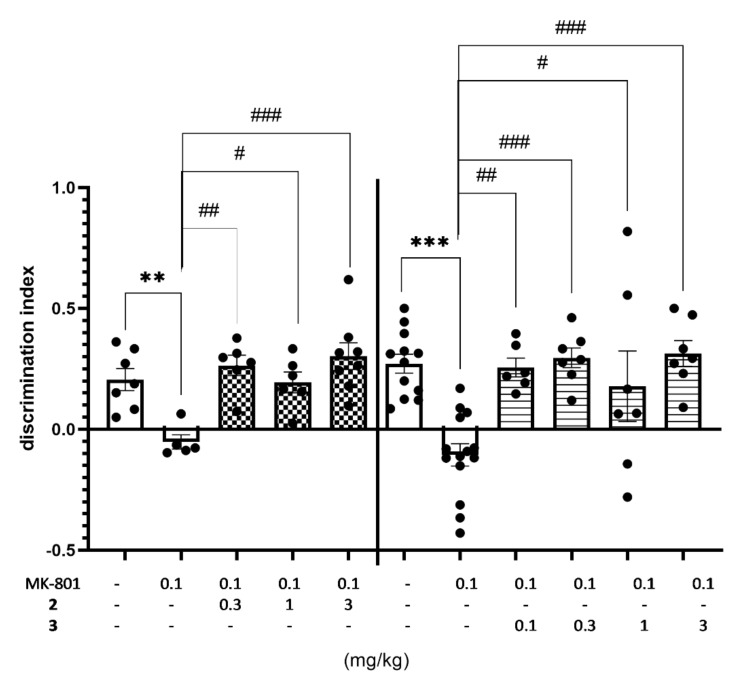
Effects of compounds **2** and **3** on MK-801-induced memory deficits in rats’ NOR test. Compounds **2** and **3** were administered i.p. 60 min, while MK-801 was given i.p. 30 min before the T1 session. The rats were observed for 3 min. The data are presented as the mean ± SEM of 6–8 rats. The data were statistically evaluated by one-way ANOVA followed by Bonferroni’s post-hoc test, ** *p* < 0.01, *** *p* < 0.001 vs. respective vehicle-treated group, and # *p* < 0.05, ## *p* < 0.01, ### *p* < 0.001 vs. respective MK-801-treated group, (one-way ANOVA for discrimination index for NOR test: F(4,27) = 7.5715, *p* < 0.001 (for compound **2**) and F(5,47) = 8.1658, *p* < 0.0001 (for compound **3**). Details shown in Supplementary Table S4.

Compounds **2** and **3** displayed the excellent ability to reverse memory impairments in this study. The discrimination index (DI) was used to reflect the preference of rats to explore the novel or familiar object, hence the higher DI value denotes the ability to reverse MK-801-induced memory impairment in NOR test (Figure 11, Table S4 in Supplementary Materials).

Moreover, the minimal effective dose of compounds **2** was as low as 0.3 mg/kg, while even lower (0.1 mg/kg) in the case of **3**. Thus, the effective dose in this test was in 10 (**2**) to 30 (**3**) fold lower than that obtained previously for compound **1** (3 mg/kg) [21]. It is worth noting that both compounds (**2** and **3**) were effective at doses distinctly lower than those of potent 5-HT_6_R agonists [10,11] and antagonists [12,13] described previously (Figure 1).

The total exploratory time of objects in the recognition phase (T2) was measured to avoid false positive results in NOR test connected with the effect of treatment on behavioral parameters. The obtained results seem to be specific hence no compound treatment increased the total exploratory time measured during T2 trial, and slight decrease of total activity was observed for compound **2** injected at the dose of 1 and 3 mg/kg jointly with MK-801 (Table 9).

#### 2.4.2. Antidepressant-Like Activity of Compounds **1**–**3** in the FST

**Figure 12:**
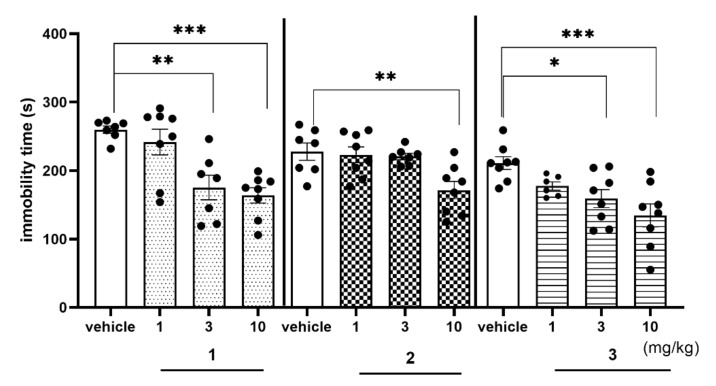
Effects of compounds **1**, **2**, and **3** on the rats’ immobility time in the FST. Compounds **1**, **2** and **3** were administered i.p. 60 min, before the test. The rats were observed for 5 min. The data are presented as the mean ± SEM of 6–8 rats. Geometrical figures represent obtained raw data; the symbol shapes for all raw data were standarized for all doses and compounds. The data were statistically evaluated by one-way ANOVA followed by Bonferroni’s post-hoc test, * *p* < 0.05, ** *p* < 0.01, *** *p* < 0.001 vs. respective vehicle-treated group, (one-way ANOVA for immobility time for FST: F(3,26) = 10.6150; *p* < 0.001 (for compound **1**), F(3,26) = 5.8561, *p* < 0.01 (for compound **2**) and F(3,26) = 6.8104, *p* < 0.01 (for compound **3**)). Details shown in Supplementary Table S5.

FST is the screening test to assess antidepressant-like properties of novel synthesized compounds. Compounds **1** and **3** showed similar antidepressant-like activity decreasing immobility time by about 30% at the dose of 3 mg/kg and by about 36% at the dose of 10 mg/kg, vs. respective vehicle-treated group both (Figure 12, Table S5, Supplementary Materials). Compound **2** also showed some antidepressant-like activity (immobility decreased by 25%), but only at the highest (10 mg/kg) dose used. The observed antidepressant-like activity of investigated compounds was distinctly weaker than the ability to reverse MK-801-induced memory impairment and may be concerned as an add-on effect to the main potential procognitive activity of the 1,3,5-triazine derivatives.

#### 2.4.3. Anxiolytic-Like Activity of Compounds **1**–**3** in the EPM test

**Figure 13:**
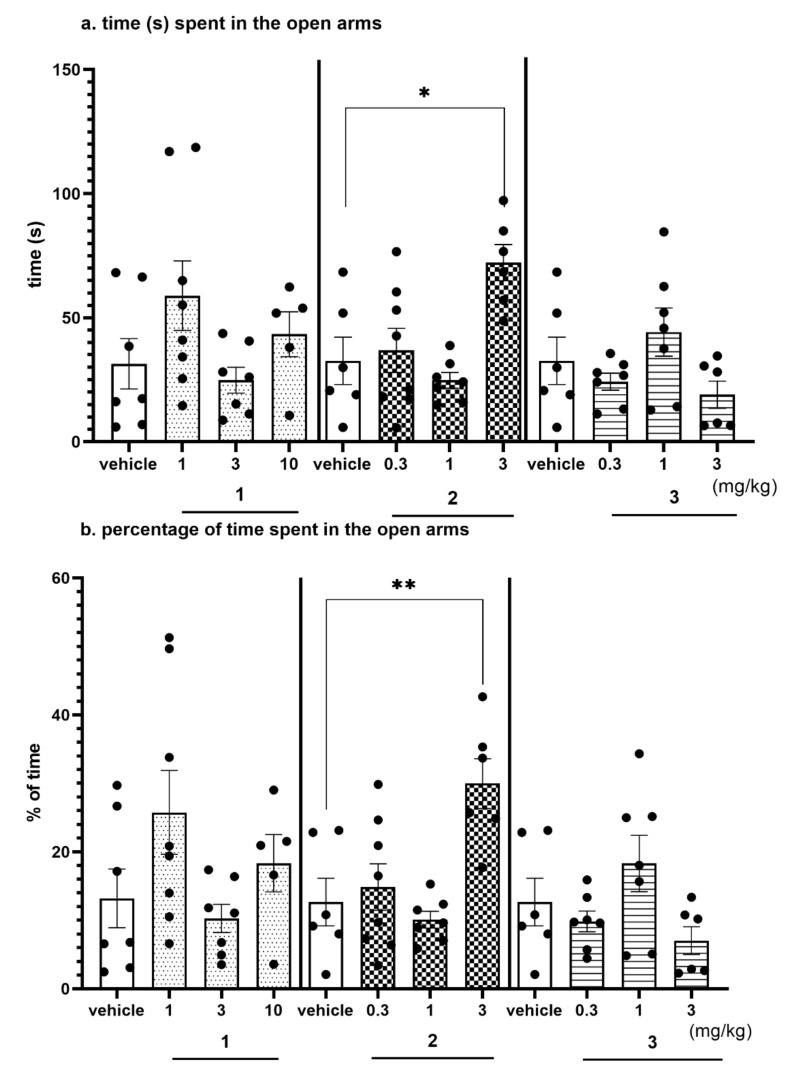

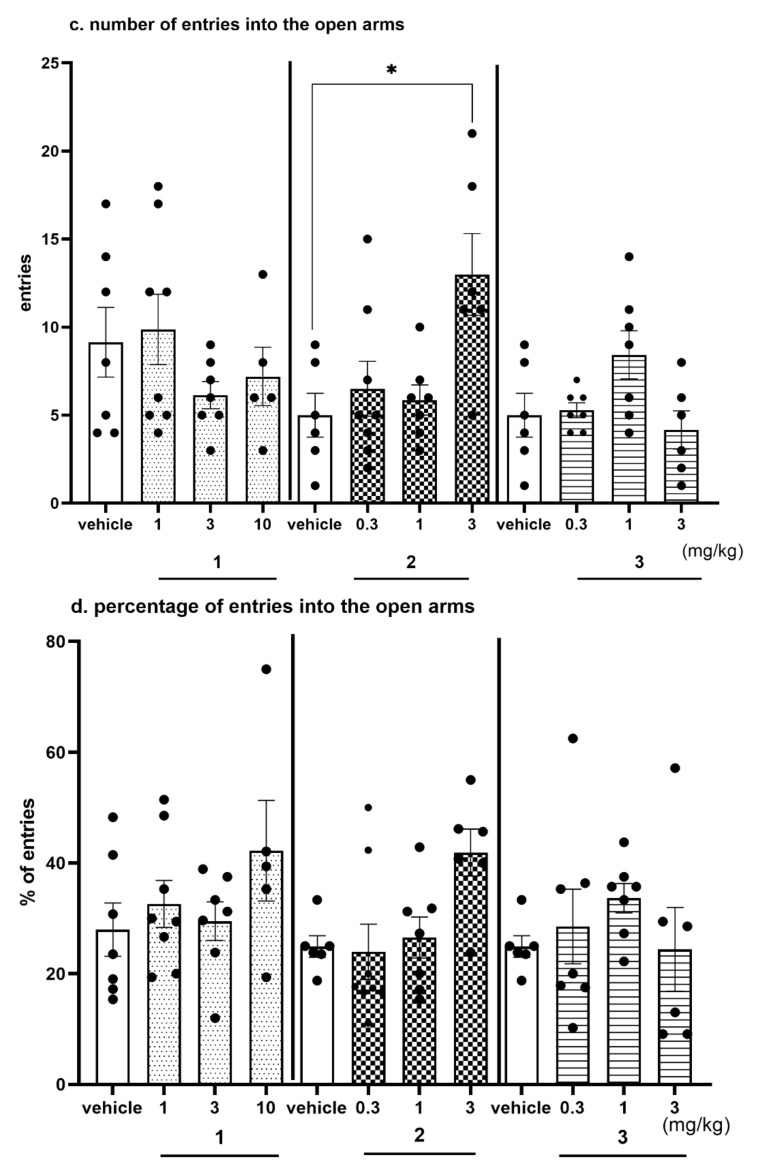

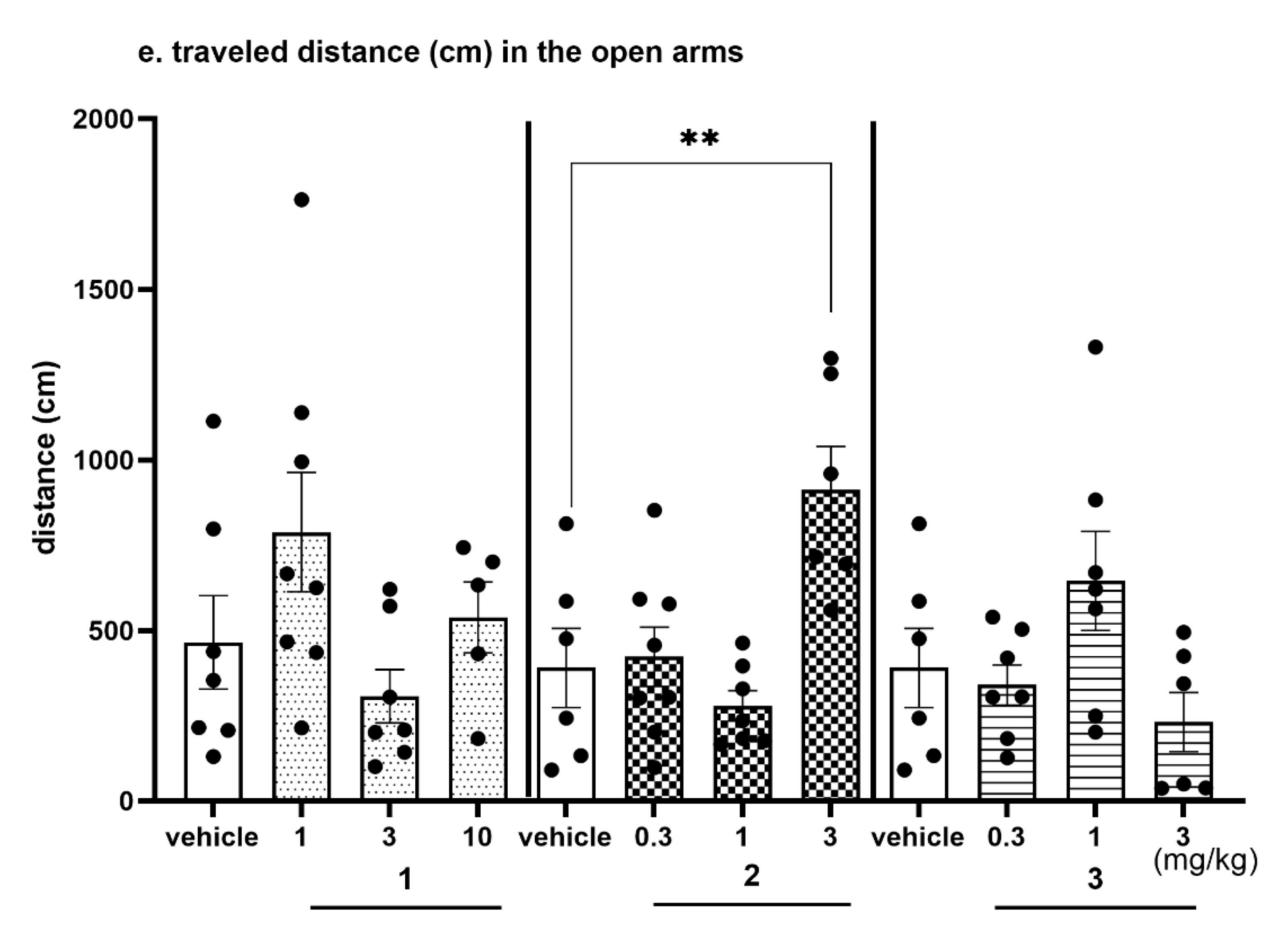
Anxiolytic-like effects of compounds **1**, **2** and **3** in the EPM test. Increased open-arm exploration denotes reduced anxiety. Compounds **1**, **2** and **3** were given i.p. 60 min, before the test. Geometrical figures represent obtained raw data; the symbol shapes for all raw data were standarized for all doses and compounds. (**a**) Time (s) spent in the open arms. (**b**) Percentage of time spent in the open arms. Values represent the mean ± SEM of the time (**a**) and percentage of time (**b**) spent in the open arms during 5-min test session compared to the respective vehicle group * *p* < 0.05, ** *p* < 0.01, (**c**) Number of entries into the open arms. (**d**) Percentage of entries into the open arms. Values represent the mean ± SEM of entries (**c**) and percentage of entries (**d**) into the open arms during 5-min test session compared to the respective vehicle group * *p* < 0.05, ** *p* < 0.01, (**e**) Travelled distance on the open arms. Values represent the mean ± SEM of the travelled distance on the open arms during 5-min test session compared to the respective vehicle group ** *p* < 0.01, (ANOVA is followed by the Bonferroni’s post hoc test); *N* = 6–8; details shown in Supplementary Table S6.

All compounds **1–3** were investigated on their anxiolytic-like activity in EPM test (Figure 13a–e, Table S6, Supplementary Materials). 

The EPM test is a well-established classical model of anxiety in rodents [36]. This test measures the ethological response to an unconditioned situation based on unlearned fear/avoidance behavior, especially rodents’ natural aversion to heights and open space. In the EPM test anxiolytic-like activity was observed only for compound **2** given i.p. at the dose of 3 mg/kg (Figure 13a–e, Table S6, Supplementary Materials). This activity was denoted by increased open arm exploration i.e., increased significantly the time spent in the open arms (Figure 13a, by 120%; ANOVA: F(3,23) = 6.9314, *p* < 0.01), percentage of time spent in open arms (Figure 13b, by 137%; ANOVA: F(3,23) = 7.7627, *p* < 0.001), the number of open-arm entries (Figure 13c, by 160%; ANOVA: F(3,23) = 5.0274, *p* < 0.01) and the distance travelled in the open arms (Figure 13d, by 134%; ANOVA: F(3,23) = 8.2188, *p* < 0.001) compared to the respective vehicle-treated group. The percentage of entries into open arms was also increased (by 68%); however, the result did not reach the significant level (Figure 13e, ANOVA: F(3,23) = 3.9489, NS). The obtained anxiolytic-like properties of compound **2** may be a desired pharmacological effect added to its basic procognitive activity assessed in NOR test. Compounds **1** and **3** did not show anxiolytic-like activity in this test.

#### 2.4.4. The Exploratory Activity of Investigated Compounds Measured in the EPM Test

All parameters presented in Table 10 describe the exploratory activity of rats that were measured using the automated version of the EPM, simultaneously with anxiolytic-like activity. There were no significant effects observed for compounds **2** and **3** in the whole dose range used (Table 10). Hence, the anxiolytic-like activity of compound **2** observed in EPM test (Figure 13a–e) is likely to reflect specific activity that cannot be explained by competing behaviors, such as the enhancement of general locomotor activity. Compound **1** at the highest dose of 10 mg/kg i.p. (but not at doses of 1 and 3 mg/kg) statistically decreased only one of the observed parameters: total distance (by 20%) which may be connected with the slightly sedative activity of this compound at the dose used (Table 10). 

## 3. Discussion

According to the chosen goal, the studies have provided a deep insight into the pharmacological and drug-likeness profiles of two highly active triazine-derived 5-HT_6_R agents (**2**, **3**) in comparison with their 2,5-dichlorophenyl analogue (**1**) partly investigated within our previous studies [21]. The achieved results allow us to discuss some qualitative structure–activity relationship (SAR) for the compounds with respect to their structural differences, i.e., *(i)* the unsubstituted phenyl moiety (**3**) and the substituted one with chlorine atoms at positions *ortho* and *meta*, in the following topology: *(ii)* 2 and 3 (**2**) or *(iii)* 2 and 5 (**1**).

The receptor profile of the compounds (**1–3**) demonstrates that both topologies of *ortho* and *para* dichlorophenyl derivatives equally contributed to the strong binding with the main target 5-HT_6_R [17], while the antagonistic action was distinctly more potent in the case of the 2,5-dichloro-substitution. The topology seems to be even more responsible for the interaction with the elected off-targets, in particular, the 5-HT_1A_R, where the affinity was almost in 10-fold stronger for the 2,3-dichlorophenyl derivative (**2**), while twice in the case of the 5-HT_2A_R (Table 1). In the latter case, the position of chlorines seems to be crucial for the intrinsic activity observed in the functional assays. Thus, the 2,3-dichlorophenyl moiety (**2**) resulted in the strong antagonism for 5-HT_2A_R, whereas the 2,5-dichloro one (**1**) provided the partial agonist mode of action (Table 2). The subtle structural differences between **1** and **2** even more influenced their ADMET profile in vitro, which could be also translated into the different behavioral activities in rats. In this context, the 2,3-dichloro-substituted derivative (**2**) almost outclassed its 2,5-dichlorsubstituted analogue (**1**) due to the much better metabolic stability, and neither hepatotoxic effect nor the DDI risk. In addition, the 2,3-dichloro derivative (**2**) proved (i) a higher ability to penetrate artificial membranes via passive transport (PAMPA), and the results correlate with those obtained in cellular-based assay (Caco-2), (ii) a better procognitive effect, i.e., this found at the dose in 10-fold lower than that of **1**, in the NOR test, and (iii) the exceptional in this group (**1**–**3**) anxiolytic-like activity in rats. Although the 2,5-dichloro-derivative (**1**) was ahead of its 2,3-dichloro analogue in terms of antidepressant effects in vivo, the much weaker ADMET profile in vitro disqualified compound **1** from in vivo pharmacokinetic studies, in the next step.

On the other hand, the unsubstituted phenyl ring (**3**) favors the selective action on 5-HT_6_R with a low risk of side effects via interactions with the receptors 5-HT_1A_, 5-HT_2A_, 5-HT_7_ and D_2_. In contrast to **1** and **2**, the absence of chlorine substituents in **3** is associated with an inability to form halogen bonds and weaker lipophilic properties. The first trait may explain a weaker affinity of **3** for 5-HT_6_R and the GPCR off-targets, in comparison with the dichlorophenyl analogues (**1** and **2**), taking into account the contribution of halogen bonds to interactions with various GPCRs. This relevance was indicated by previous lines of evidence [17]. Furthermore, the absence of chlorines (**3**) may be also responsible for less, than those of **1** and **2**, susceptibility for biotransformation, resulting with the outstanding metabolic stability of **3** (Figure 4). In turn, the decrease in lipophilicity may explain the relatively weakest ability of **3** to penetrate biological membranes (**3** vs. **1** and **2**, Table 3). It should be emphasized, however, that this is still the high capacity of **3** to cross membranes, in the range of the highly permeable CFN. Interestingly, the presence of two chlorines in the triazine derivatives **1**–**3** does not seem to be as influential as their position for ADMET properties evaluated in vitro. In most ADMET assays, a discrepancy of properties between the unsubstituted compound **3** and a chlorine derivative (**1** or **2**) was smaller than that between both chlorine derivatives (**1** and **2**), e.g., DDI risk (**1** and **3** vs. **2**, Table 6), hepatotoxic effects (**2** and **3** vs. **1**, Table 7). Results of ADMET studies in vitro have confirmed beneficial role of both, the dichloro- and unsubstituted phenyl moiety for the desirable drug-like profile, and allowed to summarize the order of drug-likeness increase, as follows: 2,5-dichlorophenyl << unsubstituted phenyl ≤ 2,3-dichlorophenyl.

The satisfying in vitro drug-like properties of 2,3-dichlorophenyl (**2**) and phenyl (**3**) derivatives were also reflected in in vivo tests. 

The main challenge of this study was to find compounds promising for an innovative AD treatment. In this context, compounds **1**–**3**, demonstrated a significant ability to reverse MK-801 induced memory impairment in NOR test in rats, and the active doses of **2**, and especially of **3**, were exceptionally low compared to the literature reports [15,37,38,39,40]. In fact, it is difficult to find either agonist or antagonist for 5-HT_6_R which reversed memory impairments in the NOR test in doses lower than 1 mg/kg at i.p. administration, while the 2,3-dichlorophenyl derivative (**2**) showed such effects at 0.3 mg/kg, and the unsubstituted phenyl derivative (**3**) even at 0.1 mg/kg i.p. The beneficial effect of 5-HT_6_ receptors ligands on memory functions has been repeatedly reported in literature. More consistent results were obtained for 5-HT_6_R antagonists that were investigated in animal models of cognitive disorders. 5-HT_6_R antagonists were shown to be effective in paradigms of episodic (NOR test) and spatial working memory (mazes or spontaneous alternation tasks), social cognition, and executive functions (set-shifting or reversal learning tasks) and in preventing memory impairments induced by scopolamine, phencyclidine (PCP), MK-801, ketamine, streptozotocin, as well as age-associated impairments (reviewed in [15,41,42,43,44]). The mechanism underlying the procognitive action of 5-HT_6_R ligands still remains unclear. Published data indicate the regulatory role of 5-HT_6_R on multiple neurotransmitter systems, and several possibilities exist. For example, 5-HT_6_R agonists are postulated to activate 5-HT_6_R located directly on cholinergic and/or glutamatergic neurons, leading to increase in cholinergic and glutamatergic transmission, while 5-HT_6_R antagonists probably act via 5-HT_6_R present on GABAergic interneurons, causing an indirect decrease in inhibitory neurotransmission, followed by enhanced cholinergic and glutamatergic function. On the other hand, autoradiography and immunohistochemistry [26] studies indicated little expression of 5-HT_6_R on cholinergic and glutamatergic neurons. On the basis of all data regarding localization of 5-HT_6_R and data from releasing experiments, it can be suggested that 5-HT_6_R agonists/antagonists modulate cholinergic or glutamatergic systems (or both) via disinhibition of GABAergic neurons. Moreover, the last literature date indicates that it is important not to forego efforts to improve also the symptoms in patients at various stages of the AD: from Mild Cognitive Impairment (MCI) to late-stage AD via mechanisms that are not necessarily directly involved in the pathological foundations of this disease [15]. Hence in our carried out behavioral studies, we also assessed potential antidepressant- and anxiolytic-like activity of compounds **1**–**3** in FST and EPM test, respectively. The obtained results indicate that these compounds possess some antidepressant-like activity in FST but minimal effective doses are 30 to 100 fold higher, respectively, than doses which can reverse MK-801 induced memory impairments. Hence this antidepressant-like activity cannot be considered as the main goal but potential additional desirable properties of compounds **1**–**3**. Moreover only for compound **3** some anxiolytic-like activity in EPM test was assessed. The minimal active dose of compound **3** in this test was 30 fold higher than minimal active dose in NOR test. 

The presented behavioral results indicate that investigated 1,3,5-triazine derivatives are more potent to reverse memory impairments and may possess some additional central activities. However in this paper we present only preliminary behavioral data which may be extended in the future, e.g., another model of memory impairment or chronic administration of the most interesting compounds given alone or jointly with e.g., donepezil. The reason of these studies was to assess the pharmacological activity, especially potential procognitive activity of 1,3,5-triazine derivatives but the most interesting for us at this stage of investigations was to compare and find possible relationships between pharmacodynamic and pharmacokinetic properties assessed in vivo as well as in vitro ADMET investigations. The obtained results may board the knowledge about these compounds for further studies. Interestingly, the procognitive activity of the compounds does not correlate with their 5-HT_6_R affinities. Intriguingly, the highest procognitive activity was shown by compound **3** with the relatively lowest affinity for the 5-HT_6_R. This is even more surprising as the weak affinity of this compound for the remaining GPCRs excludes eventual multitarget effects that would be probable to support the action of compounds **1** and **2**. The significantly more potent procognitive effects of 2,3-dichlorophenyl derivative (**2**) than that of 2,5-dichlorophenyl one (**1**) can be explained based on both, distinctly higher metabolic stability (80% of 2 vs. < 10% of 1, Figure 4) and slightly better permeability (20.5 for 2 vs. 18.9 for **1**, Table 3) of **2**. In general, the low metabolic stability of **1** suggests that all its pharmacological effects in vivo, confirmed within this and previous studies [17], are caused by either **1** or its metabolites, or even predominantly by its metabolites. This hypothesis can partly explain the stronger antidepressant-like action of **1** than that of **2**, due to a likely incorporation of active metabolite(s) of **1** into the action. As an additional explanation, the relevant difference between **1** and **2** in the binding and functional profiles towards the off-targets (especially 5-HT_2A_R) can be mentioned. The drug-like profile of **1**, differing and rather worse than that of **2** and **3**, is able to justify in general terms the better procognitive action observed for the latter two, despite the strongest 5-HT_6_R antagonistic action of **1** (Table 2). In contrast, the procognitive potency of **3**, higher than that of **2**, needs a wider analysis, taking into account that **2** not only showed more potent 5-HT_6_R antagonistic activity, but also the better membrane permeability (20.5 for 2 vs. 15.1 for 3, Table 3), and the metabolic stability only slightly lower (80% for 2 vs. 95.7% for 3, Figure 4). Results of our comprehensive pharmacokinetic study in vivo give some support in this question. 

The aim of the pharmacokinetic studies in vivo was to estimate basic pharmacokinetic parameters of the 2,3-dichlorophenyl compound (**2**) and the unsubstituted phenyl derivative (**3**) in rats, after administration of their common active dose determined in behavioral NOR test (0.3 mg/kg i.p.). Results indicated that both compounds **2** and **3** had a beneficial pharmacokinetic profile. However, there were some differences that could be crucial for the action observed in the behavioral studies. Thus, after i.p. administration of both **2** and **3**, the absorption was relatively rapid, but compound **3** was detected in serum from the first blood sampling time (i.e., 5 min) and rapidly reached T_max_ (5 min) compared to compound **2** which reached C_max_ slightly later (T_max_ = 15 min) for the dose studied. The apparent volume of distribution (Vz/F) during terminal phase for compound **2** (81 L/kg) was significantly higher (*p* < 0.05) as compared to compound **3** (65 L/kg), what might indicate its stronger bound by rat tissues. Both compounds **2** and **3** were also characterized by a favorable value of serum elimination half-life (t0.5λz), but were almost 2-fold longer in the case of the 2,3-dichlorophenyl compound (116 min vs. 77 min, Table 7). The values of this parameter for brain tissue were even higher and it was 149 min for 2, and 112 min for **3**. 

Tissue distribution of compounds **2** and **3** were assessed also after their i.p. administration at a dose of 0.3 mg/kg to rats. The highest concentration of **2** and **3** in the brain (C_max_) was observed after 15 and 5 min, respectively, which may indicate a rapid distribution of these compounds to the target tissue. It is worth noting that the concentrations of **2** and **3** in the brain tissue were higher than in the serum in the entire sampling interval of these compounds (5–240 min). A relatively high value of K_p_ was also observed in the brain both for compound **2** and **3** (2.03 vs. 2.63, *p* > 0.05), indicating that these compounds are well distributed to this organ which is in line with the central activity of these compounds observed in behavioral studies. The beneficial pharmacokinetics of both compounds in vivo is in a high concordance with their ADME profile in vitro, thus underlining very promising properties of a potential CNS-drug candidate for either **2** or **3**. 

In order to solve the riddle of a more potent procognitive potential of **3** comparing to **2** found in the NOR test, we also carried out a detailed analysis of the time-concentration changes of both compounds (**2** and **3**) in brain during 240 min, with particular emphasis on the first 60 min corresponding with the time of NOR test. Thus, a distinctly higher concentration of compound **3** compared to **2** was seen in the first 30 min (Figure 9). This may be the reason that the compound **3** was able to reach the necessary “therapeutic concentration” at a lower dose administrated than that of **2** in the conditions simulating memory disorders typical for the NOR test. While this fact may explain the differences in the activities of the two compounds (**2** and **3**) observed in the NOR test, it does not prejudge a predominant therapeutic potential of **3**, with respect to **2**, in the treatment of memory disorders. The full observation indicated the almost 2-fold higher concentration in the brain for compound **2** compared to **3** maintained from 60 to 240 min. This, combined with a better receptor profile (strong 5-HT_6_R and 5HT_2A_R antagonism), can even point the 2,3-dichlorophenyl derivative **2** as a potentially better CNS drug candidate, also due to a slightly better in vitro safety profile (lower risk of DDI) than that of **3**.

Summing up, the reduced SAR analysis, on the basis of RBA, functional assays, the comprehensive ADMET in vitro and both, pharmacokinetic and behavioral in vivo studies, indicates beneficial properties of a potential CNS-drug candidate useful against dementia disorders for both (*RS*)-4-[1-(2,3-dichlorophenoxy)propyl ]-6-(4-methylpiperazin-1-yl)-1,3,5-triazin-2-amine (**2**) and (*RS*)-4-(4-methylpiperazin-1-yl)-6-(1-phenoxypropyl)-1,3,5-triazin-2-amine (**3**), with slightly predominant pharmaceutical profile for the 2,3-dichlorophenyl derivative **2**.

## 4. Materials and Methods

### 4.1. Compounds

Compounds **1–3**, i.e., (RS)-4-[1-(2,5-dichlorophenoxy)propyl ]-6-(4-methylpiperazin-1-yl)-1,3,5-triazin-2-amine (**1**), (RS)-4-[1-(2,3-dichlorophenoxy)propyl ]-6-(4-methylpiperazin-1-yl)-1,3,5-triazin-2-amine (**2**) and (RS)-4-(4-methylpiperazin-1-yl)-6-(1-phenoxypropyl)-1,3,5-triazin-2-amine (**3**) were synthesized at Department of Technology and Biotechnology of Drugs. Full description of investigated derivatives was reported previously [21]. Their purity and identity was confirmed using NMR spectroscopy and LC-MS. All compounds were submitted as basic forms and racemates and characterized by high purity **1**–100%, **2**–100%, **3**–96% (LC-MS). 

### 4.2. Radioligand Binding Assay

The cells used in the radioligand binding assays receptor 5-HT_1A_ were stably expressed in HEK-293 cells. Transfections were performed with the use of Lipofectamine 2000. The binding procedure was accomplished via the displacement of 2.5 nM [^3^H]-8-OHDPAT (135.2 Ci/mmol) for 5-HT_1A_R. Each compound was tested in triplicate in seven to eight different concentrations (10^−11^ to 10^−4^ M). The inhibition constants (*K_i_*) were calculated using the Cheng–Prusoff equation [45] and the results are expressed as the mean of at least two independent experiments.

### 4.3. Functional Assay

#### 4.3.1. Functional Assays for 5-HT_6_ Receptor

Test compounds were dissolved in dimethyl sulfoxide (DMSO) at a concentration of 10 mM. Serial dilutions were prepared in 96-well microplate in assay buffer and eight concentrations were tested. For the 5-HT6, adenylyl cyclase activity were monitored using cryopreserved 1321N1 cells with expression of the human serotonin 5-HT_6_ receptor (Perkin Elmer, Waltham, MA, USA). Thawed cells were resuspended in stimulation buffer (HBSS, 5 mM HEPES, 0.5 IBMX, and 0.1% BSA at pH 7.4) at 3 × 10^5^ cells/mL. The same volume (10 μL) of cell suspension was added to tested compounds. Samples were loaded onto a white opaque half area on a 96-well microplate. The antagonist response experiment was performed with 11 nM serotonin as the reference agonist for 5-HT6 receptor. The agonist and antagonist were added simultaneously. Cell stimulation was performed for 30 min at room temperature. After incubation, cAMP measurements were performed with homogeneous TR-FRET immunoassay using the LANCE Ultra cAMP kit (PerkinElmer, Waltham, MA, USA). 10 μL of EucAMP Tracer Working Solution and 10 μL of ULight-anti-cAMP Tracer Working Solution were added, mixed, and incubated for 1 h. The TR-FRET signal was read on an EnVision microplate reader (PerkinElmer, Waltham, MA, USA). IC 50 and EC 50 were determined by nonlinear regression analysis using GraphPad Prism 7.0 software (GraphPad Software, La Jolla, CA, USA).

#### 4.3.2. Functional Assays for 5-HT_2A_ Receptor

Test compounds were dissolved in dimethyl sulfoxide (DMSO) at a concentration of 10 mM. Serial dilutions were prepared in a 96-well microplate in assay buffer and eight concentrations were tested. A cellular aequorin-based functional assay was performed with recombinant CHO-K1 cells expressing mitochondrially targeted aequorin, human GPCR and the promiscuous G protein α16 for 5-HT_2A_. The assay was executed according to the previously described protocol [46]. After thawing, cells were transferred to assay buffer (DMEM/HAM’s F12 with 0.1% protease-free BSA) and centrifuged. The cell pellet was resuspended in assay buffer and coelenterazine h was added at final concentrations of 5 μM. The cells suspension was incubated at 16 °C, protected from light with constant agitation for 16 h and then diluted with assay buffer to the concentration of 250,000 cells/mL. After 1 h of incubation, 50 μL of the cells suspension was dispensed using automatic injectors built into the plate reader POLARstar Omega, (BMG Labtech, Ortenberg, Germany) onto a white opaque 96-well microplates preloaded with test compounds. Immediate light emission generated following calcium mobilization was recorded for 30 s. In antagonist mode, after 30 min of incubation, the reference agonist was added to the above assay mix and light emission was recorded again. The final concentration of the reference agonist was equal to EC80.

### 4.4. ADMET In Vitro 

#### 4.4.1. References

The compounds used as the references: caffeine (CFN), doxorubicin (DX), ketoconazole (KE) and nonyl-4-hydroxyquinoline-N-oxide (NQNO) were obtained from Sigma-Aldrich (St. Louis, MO, USA). 

#### 4.4.2. Permeability

The 96-well Pre-coated PAMPA Plate System Gentest™ that was used was obtained from Corning (Tewksbury, MA, USA). The tested 5-HT_6_R ligands and the reference (CFN) solutions (**1,3** at 200 µM and **2** at 100 µM) were prepared in PBS buffer (pH = 7.4) and added to the donor wells (300 μL/well). 200 μL/well of PBS was added to the acceptor wells. All compounds were analyzed in triplicate. The plate was incubated at room temperature for 5 h. Then, the 50 μL was aspirated from each well and diluted next with 50 µL solution of an internal standard (IS). The compounds’ concentrations in acceptor and donor wells were estimated by the UPLC-MS analyses, which were performed by LC/MS Waters ACQUITY™ TQD system with the TQ Detector (Waters, Milford, CT, USA). The permeability coefficients (Pe, cm/s) were calculated according described previously formulas [24,27,28]. According to the PAMPA plate’s manufacturer, compounds with Pe values higher than 1.5 × 10^−6^ cm/s possess good human oral absorption capacity [22].

#### 4.4.3. Caco-2 Permeability Assay

Caco-2 cells were purchased from the American Type Culture Collection (ATCC^®^ no. HTB37™) and were cultured in the Dulbecco’s modified Eagle’s Medium supplemented with 10% heat-inactivated fetal bovine serum and 1% nonessential amino acid solution. The cells were grown in a T-75 flask in a humidified atmosphere of 5% CO_2_/95% at 37 °C. Confluent monolayers were subcultured by treatment with 0.25% trypsin and 0.2% EDTA and the cell suspension was gently passed through a 27-gauge needle. For the permeability study, Caco-2 cells of passage numbers 22–26 were used. The cells were seeded in transwell inserts (polycarbonate membrane, 6.5-mm diameter and 0.4-μm pore size, Corning Costar Co., Tewksbury, MA, USA) in 24-well plates at a density of 1.65 × 10^4^ cells/insert (0.33 cm^2^/insert). The transwell inserts were pre-wet with a complete growth medium for 15 min before seeding. The basolateral and apical compartments contained 0.3 and 0.6 mL of culture medium, respectively. One insert was cultivated without cells since this was required for the measurement of the TEER (transepithelial electrical resistance) blank value. The medium was changed after 12 h post-seeding to avoid multilayer formation. Then the culture medium was replaced three times a week until the end of the cultivation period on day 21. On the day of the transport experiment, the medium from transwell inserts was decanted and the inserts were transferred into a new 24-well plate containing warmed Hanks’ balanced salt solution (HBSS), pH 7.4. HBSS was carefully added to the apical side. The inserts were washed for 30 min in a CO_2_ incubator under gentle shaking (150 r.p.m.). To evaluate the integrity of the cell monolayer, TEER was measured using the Millicell ER-2 (Millipore, Billerica, MA, USA). For experiments in our laboratory, we only used monolayers on inserts with a TEER value of ≥ 250 Ω cm^2^ at 37 °C. The integrity of the cells was checked before and after the experiment by measuring the TEER. The transport experiment was performed in either the apical to the basolateral direction (A->B, for passively transported compounds) or the basolateral to the apical direction (B->A, for actively transported compounds). According to this pattern, the tested compounds were diluted in HBSS to a recommended concentration of 10 µM and applied to the apical chamber (for A->B) or the basolateral chamber (for B->A), and HBSS was added to the other side. Caffeine as a highly permeable reference was used. The Caco-2 plate was then incubated for 2 h at 37 °C in a CO_2_ incubator under gently shaking (150 r.p.m.). The samples were taken from both apical and basolateral compartments and compounds concentration was quantified by peak area analysis on LC-MS system with internal standard (IS). Test permeability and efflux ratio for each compound was done in triplicate. To ensure monolayer integrity throughout the experimental period, lucifer yellow (LY) rejection was measured. LY is a fluorescent dye that is only transported para-cellularly and is thus used as a marker of Caco-2 tight junction integrity. After the transport experiment was completed, transwell inserts were washed, LY was added to the apical compartment at a concentration of 60 μM, whereas HBSS was added to the basolateral one. The plate was incubated for 1 h at 37 °C in a CO_2_ incubator under gently shaking (150 r.p.m.). The fluorescence of the LY transported to the basolateral side was then measured with EnSpire multiplate reader (Perkin Elmer, Waltham, MA, USA). The permeability *Papp* was calculated according to the following formula [47]:

$$P_{app}=\frac{dc}{dt}*V/\left(A*C_{0}\right)$$

*dc/dt*—the change in concentration in the receiving compartment overtime

*V*—volume of the solution in the receiving compartment (mL)

*A*—surface area of the membrane (cm^2^)

*C_0_*—the initial concentration in the donor compartment (µM)

Based on in vitro/in vivo correlation studies, *Papp* values obtained from Caco-2 assay predicts the following range of in vivo absorption: Low (0–20%): Papp ≤ 10^−6^ cm/s; Medium (21–80%): 10^−6^ cm/s < Papp ≤ 10 × 10^−6^ cm/s; High (81–100%): Papp > 10 × 10^−6^ cm/s [48]. 

#### 4.4.4. Metabolic Stability

The metabolic stability parameters of compounds **2–3** were estimated by using rat liver microsomes (RLMs) (Sigma-Aldrich, St. Louis, MO, USA), according to the described previously protocols [24,27,28]. The in vitro evaluation of metabolic pathways was performed by prolonged, 120 min incubation of compounds **2,3** with RLMs. The tested 5-HT_6_R ligands (50 µM) were incubated in the presence of RLMs (1 mg/mL) in 10 mM Tris–HCl buffer (37 °C). The LC/MS analyses with additional MS ion fragmentation of the products and substrates were performed to determine the most probable structures of 5-HT_6_R ligands’ metabolites. LC/MS Waters ACQUITY™ TQD system with the TQ Detector (Waters, Milford, CT, USA). 

The in silico prediction of metabolic pathways was performed by MetaSite 8.0.1 software (Molecular Discovery Ltd., Hertfordshire, UK). The computational liver model of metabolism was used to determine the most probable sites of biotransformation and identify the structures of obtained in vitro metabolites.

#### 4.4.5. Safety

The luminescent CYP3A4 P450-Glo™ Promega^®^ (Madison, WI, USA) was used for the investigation of potential drug-drug interactions. All assays and protocols were described before [24,27,28]. The compounds were tested in triplicate at the final concentrations in the range from 0.01 to 25 μM. The luminescent signal was measured by using a microplate reader EnSpire PerkinElmer (Waltham, MA, USA).

The hepatotoxicity of compounds **2–3** were evaluated with the use of *hepatoma* HepG2 (ATCC^®^ HB-8065™). Cells were grown under previously described conditions [24,27,28]. Compounds **2–3** were incubated on a 96-well plate with cells for 72 h in the final concentration range (0.1–100 μM), whereas the reference DX at 10 μM. The cells’ viability was determined by CellTiter 96^®^ AQueous Non-Radioactive Cell Proliferation Assay (MTS), which was purchased from Promega^®^ (Madison, WI, USA). The absorbance was measured using a microplate reader EnSpire (PerkinElmer, Waltham, MA USA) at 490 nm. All compounds were tested in quadruplicate. 

The mutagenicity of 5-HT_6_R ligands **1–3** was evaluated by Ames microplate fluctuation protocol (MPF) obtained from Xenometrix, (Allschwil, CHE). The used *Salmonella* Typhimurium TA100 strain has base pair substitution (hisG46 mutation, which target is GGG). The experiments were performed as described before [24,27,28]. The compounds **1–3** were tested in two final concentrations, 1 and 10 μM, in triplicate. The occurrence of revertants was visualized by pH indicator dye which was present in the bacterial medium. The color changes from violet to yellow were visually counted and confirmed by measurements of absorbance with a microplate reader (EnSpire) at 420 nm.

#### 4.4.6. Neurotoxicity

Human neuroblastoma cell line SH-SY5Y (ATCC^®^ no. CRL-2266™) was used for neurotoxicity evaluation. The cells (2 × 10^4^ cells/200μL/well) were cultured in transparent 96-well plates (Nunc) in DMEM/F12 supplemented with 10% FBS in the presence of dimethylsulfoxide (DMSO < 0.1%, vehicle control) or increasing concentration of compounds **1, 2** and **3** (4.9 × 10^−8^–1 × 10^−4^ M). To perform dose–response analysis, 2-fold serial dilutions (12 points) were prepared. Treatment with compounds was performed for 48 h. After the incubation time, 100μL of culture medium was transferred to black well plates to examined membrane integrity using the CytoTox-ONE™ Homogeneous Membrane Integrity Assay (Promega, Madison, WI, USA) following the manufacturer’s protocol. Briefly, a volume of 100 µL CytoTox-ONE™ reagent was added to each well and carefully mixed under gentle shaking. 4 µL of lysis solution (Promega, Madison, WI, USA) were added to the wells containing the control cells (maximum LDH release control) for 5 min after which 100 µL of lysis solution was also transferred to black well plates and 100 µL CytoTox-ONE™ reagent was added. The plate was incubated at room temperature for 15 min and the fluorescence was then recorded at an excitation wavelength of 560 nm and an emission wavelength of 590 nm using the microplate reader EnSpire (PerkinElmer, Waltham, MA, USA). To calculate the percentage cytotoxicity, the average fluorescent values of the background medium were subtracted from all average fluorescent values of the experimental samples and the positive control. Cytotoxicity is expressed as a percentage relative to the positive control, which is assumed to be responsible for 100% LDH release.

The cell viability was examined using an MTS-based [3-(4,5-dimethylthiazol-2-yl)-5-(3-carboxymethoxyphenyl)-2-(4-sulfophenyl)-2 H tetrazolium] CellTiter96^®^ AQueous One Solution Cell Proliferation Assay (Promega, Madison, WI, USA) following the manufacturer’s protocol. Briefly, 20 μL of MTS solution was pipetted into each well containing 100 μL of culture or culture medium (negative control) and incubated at 37 °C for 1.5 h. After incubation time, formazan product turnover absorbance was measured at 490 nm using the microplate reader EnSpire (PerkinElmer, Waltham, MA, USA). Percentage cell viability was calculated as 100% × (absorbance of treated cells—absorbance of background controls)/(absorbance of DMSO vehicle controls—absorbance of background controls).

#### 4.4.7. Determination of Intracellular ROS Levels

ROS measurement was assayed by 2′,7′-Dichlorofluorescin diacetate (DCFH-DA, Sigma D6883) [49]. All treatments to the cells were achieved with warmed HBSS and during the operational steps, the cells were kept on the plate heated to 37 °C to minimalise the temperature stress. Briefly, SH-SY5Y cells (4 × 10^3^ cells/well) were seeded in a black-sided, clear-bottom 384-well plate (Life Technologies, Warsaw, Poland) in DMEM without phenol red (Life Technologies, Warsaw, Poland) supplemented with 5% FBS and cultured for 24 h. The next day, the medium was removed, the cells were washed once with HBSS and treated for 45 min with non-fluorescent dye DCFH-DA (final concentration 25 μM, freshly prepared in warm HBSS). After staining, the cells were washed once with HBSS following 1 h pretreatment with HBSS containing tested compounds 1, 2 and 3. After that, toxic agents were added: H_2_O_2_ (200 μM), oxaliplatin (OXA, 25 μM) and rotenone (ROT, 32.5 μM) and the cells were incubated for 5 h. In our preliminary experiments, different concentration of toxic agents was tested to choose the convenient one that leads to 50% cell death. As a positive control, the cells were incubated alone with toxic agents. As a vehicle control, the cells were incubated with HBSS with 0.1% DMSO. In the presence of ROS, the non-fluorescent dye DCFH-DA is oxidized, producing fluorescent dichlorofluorescein (DCF). Fluorescence was measured at Ex/Em = 485/535 nm using the microplate reader EnSpire.

### 4.5. In Vivo Pharmacokinetic Studies

#### 4.5.1. Animals

The experiments were performed on male Wistar rats (200–230 g) obtained from an accredited animal facility at the Jagiellonian University Medical College, Poland. The animals were housed in a group of four in a controlled environment (ambient temperature 21 ± 2 °C; relative humidity 50–60%; 12 h light/dark cycles (lights on at 8:00). Standard laboratory food (LSM-B) and filtered water were freely available. Animals were assigned randomly to treatment groups. For pharmacokinetic studies animals were fasted before dosing by withholding food but not water overnight. After dosing, food was withheld for an additional 8 hr. All animals were used only once. Procedures involving animals and their care were conducted following current European Community and Polish legislation on animal experimentation. Additionally, all efforts were made to minimize animals’ suffering and to use only the number of animals necessary to produce reliable scientific data. The experimental protocols and procedures described were approved by the I Local Ethics Commission in Cracow (no 309/2019, 17.07.2019) and complied with the European Communities Council Directive of 24 November 1986 (86/609/EEC) and were under the 1996 NIH Guide for the Care and Use of Laboratory Animals.

#### 4.5.2. Application to a Pharmacokinetic Study in Rats

To assess pharmacokinetic profile and tissue penetration of **2** and **3**, the male Wistar rats were a single intraperitoneal (i.p.) injected with these compounds dissolved in tween (vehicle volume 1 mL/kg) at a dose of 0.3 mg/kg, determined in behavioral studies. The animals were killed by decapitation under deep anesthesia (i.p. injection of 50 mg/kg ketamine and 7.5 mg/kg xylazine) at 5, 15, 30, 60, 120 and 240 min after compounds administration (four animals per time point) and blood samples (approximately 5–6 mL) were collected into tubes. Moreover, five tissues (i.e., brain, heart, lungs, kidneys and liver) were harvested, rinsed with cold saline, and the wet weights determined. Blood was allowed to clot for 15–20 min at room temperature and then centrifuged (3000 rpm for 10 min). The obtained serum and tissues were stored frozen at −80 °C until analysis.

#### 4.5.3. Analytical Method

The concentrations of both test compounds in serum and tissue homogenates were measured by a reverse-phase high-performance liquid chromatography method with ultraviolet detection (HPLC/UV). Samples were separated on a Supelcosil LC-PCN column (250 × 4.6 mm, 5 μm particle size) with an integrated pre-column (Sigma-Aldrich, Bellefonte, PA, USA). The mobile phase consisted of methanol/10 mM potassium dihydrogen phosphate buffer, pH 4.6/acetonitrile (51/40/9, *v*/*v*/*v*). The isocratic separation was performed at 1.0 mL/min constant flow-rate with column temperature maintained at 38 °C. The detection wavelength was set at 206 nm for **2** and at 215 nm for **3**. The HPLC system consisted of a Hitachi-Elite LaChrom L-2130 pump, with an ultraviolet-visible (UV-Vis) detector (L-2400), a LaChrom L-2300 column oven, and an L-2200 autosampler (VWR, Darmstadt, Germany). EZChrome Elite v. 3.3.2 software (VWR, Darmstadt, Germany) was used for data acquisition. 

#### 4.5.4. Determination of Compounds **2** and **3** in Biological Matrices

Before analysis, tissue samples were thawed, placed in four volumes (*w*/*v*) of phosphate-buffered saline (PBS, pH 7.4) and homogenized using a MICCRA D-1 homogenizer (ART Prozess- & Labortechnik GmbH & Co., Heitersheim, Germany). To isolate **2** or **3**, 500 µL of rat serum or tissue homogenate containing this compound were mixed with a 20-μL volume of IS solution (50 ng/mL or 200 ng/g in methanol). As an IS for compound **2** quantification, compound **3** was used and vice versa.

The samples were alkalized with 50 μL of 4 M sodium hydroxide solution, vortex-mixed, and extracted with 1 mL of ethyl acetate/hexane (30/70, *v*/*v*) mixture on a shaker (VXR Vibrax, IKA, Legnica, Poland) for 20 min. After centrifugation (Eppendorf, Mini Spin Plus, Hamburg, Germany), the organic layers were transferred into new tubes containing 100 μL of methanol and 0.1 M sulfuric acid (10/90, *v*/*v*) mixture. Then, the samples were shaken and centrifuged again. Finally, 20–90 μL of each acidic layer was injected into the HPLC system. 

Validation of the assay was performed according to the FDA guideline (Bioanalytical method validation) [50]. This included selectivity, linearity, accuracy and precision, the limit of detection and limit of quantitation, recovery, and stability.

#### 4.5.5. Pharmacokinetic Analysis 

The peak serum compounds **2** and **3** concentration (C_max_) and its time of occurrence (T_max_) were directly read from the concentration–time data. Other pharmacokinetic parameters were determined on subjecting the concentration–time data to non-compartmental analysis using Phoenix WinNonlin 8.2 software (Pharsight Corporation, a Certara Company, Princeton, NJ, USA). 

### 4.6. Behavioral Studies In Vivo

#### 4.6.1. Animals

The experiments were performed on 254 male Wistar rats (230–260 g, 8 weeks of age) obtained from an accredited animal facility at the Jagiellonian University Medical College, Poland. The animals and during them were housed in a group of four in a controlled environment (ambient temperature 21 ± 2 °C; relative humidity 50–60%; 12 h light/dark cycles (lights on at 8:00). Standard laboratory food (LSM-B) and filtered water were freely available. One week before experiments animals were handled to acclimatize to researechers’ touch to minimize stress reaction of animals. Animals were assigned randomly to treatment groups. All the experiments were performed by two observers unaware of the treatment applied between 9:00 and 14:00 on separate groups of animals. All animals were used only once. Procedures involving animals and their care were conducted under current European Community and Polish legislation on animal experimentation. Additionally, all efforts were made to minimize animal suffering and to use only the number of animals necessary to produce reliable scientific data. The experimental protocols and procedures described in this manuscript were approved by the I Local Ethics Commission in Cracow (no 309/2019, 17.07.2019) and complied with the European Communities Council Directive of 24 November 1986 (86/609/EEC) and were under the 1996 NIH Guide for the Care and Use of Laboratory Animals. 

#### 4.6.2. Drugs

The investigated compounds **1**, **2** and **3** were suspended in 1% Tween 80 immediately before administration, while MK-801 (MK-801 maleate, Bio-Techne, Warsaw, Poland) was dissolved in distilled water. All compounds were given in a volume of 2 mL/kg. Compounds **1**, **2** and **3** were administered intraperitoneally (i.p.) 60 min while MK-801 was given i.p. 30 min before testing. Control animals received vehicle (1% Tween 80 (Sigma Aldrich, Poznan, Poland)) according to the same schedule. 

#### 4.6.3. Behavioral Procedures in Rats

##### Novel Object Recognition (NOR) Test 

Five days before the experiment, the rats were transferred to the laboratory, labelled and, thereafter, left to acclimate to the new environment. The animals were handling every five days before experiments to minimize the stress reaction. The protocol was adapted from the original work [51,52]. The test session comprising of two trials separated by an inter-trial interval (ITI) of 1 h was carried out after 2 days of the training session. During the first trial (familiarization, T1) two identical objects (A1 and A2) were presented in the opposite corners of the open field, approximately 10 cm from the walls. During the second trial (recognition, T2) one of the A objects was replaced by a novel object B, so that the animals were presented with the A = familiar and B = novel objects. Both trials lasted for 3 min and the animals were returned to their home cages after T1. 

The objects used were the metal Coca-Cola cans and the glass jars filled with sand. The heights of the objects were comparable (~12 cm) and the objects were heavy enough not to be displaced by the animals. The sequence of presentations and the location of the objects were randomly assigned to each rat. After each measurement, the floor was cleaned and dried.

The animals explored the objects by looking, licking, sniffing or touching the object but not when leaning against, standing or sitting on the object. Any rat exploring the two objects for less than 5 s within 3 min of T1 or T2 was eliminated from the study. The exploration time of the objects was measured by a blind experimenter. Based on the exploration time (E) of two objects during T2, the discrimination index (DI) was calculated according to the formula: DI = (EB − EA)/(EA + AB). Using this metric, scores approaching zero reflects no preference while positive values reflect a preference for the novel object and negative numbers reflect a preference for the familiar.

MK-801 has been chosen because it induces, by non-competitive blocking of NMDA receptors [38], cognitive disruptions similar to those associated with dementia [37] and schizophrenia [53], which were the potential therapeutic targets of 5-HT_6_R antagonists tested in clinical trials [40]. Additionally, 5-HT_6_R appears to be implicated in regulation of glutamate release (for review see [15,39]). Previously we showed that both, a selective 5-HT_6_R agonist and an antagonist, given acutely and chronically, prevent memory impairments induced by MK-801 in rats [35]. Hence, we decided in this paper to also use MK-801 as a compound to induce memory impairment in the present studies.

MK-801, used to attenuate learning, was administered at the dose of 0.1 mg/kg (i.p.) 30 min before the familiarization phase (T1), while investigated compounds were given 60 min before T1 session. In such scheme of experiment we could observe the impact of investigated compounds on the one type of rats’ memory—episodic memory and one phase of memory process—reconsolidation. 

The total exploration time in T2 was used to express the influence of the treatment on the exploratory activity of the animals.

##### Forced Swim Test (FST)

The experiment was carried out according to the method of [54]. On the first day of the experiment, the animals were gently individually placed in Plexiglas cylinders (40 cm high, 18 cm in diameter) containing 15 cm of water maintained at 23–25 °C for 15 min. On removal from water, the rats were placed for 30 min in a Plexiglas box under 60-W bulb to dry. On the following day (24 h later), the rats were replaced in the cylinder and the total duration of immobility was recorded during the whole 5-min test period. The immobility was assigned when no additional activity was observed other than that necessary to keep the rat’s head above the water. Fresh water was used for each animal. 

##### Elevated Plus-Maze Test (EPM Test) 

The testing procedure was based on a method described by [55]. Plus-maze apparatus (an automated device produced by Campden Instruments Ltd. (Leicestershire, UK) made of durable, high density, non-porous black plastic, elevated to a height of 50 cm, consisted of two open arms (50 × 10 cm) and two closed arms (50 × 10 cm, and 30 cm high walls), arranged so that the two arms of each type were opposite each other. The floor of the plus-maze was made of infrared transparent material what means that there are no visible sensors. Plus-maze apparatus was connected to PC software by control chassis. The experiments were conducted in a darkened room, only the center of the maze was illuminated with low-intensity light (30 lux measured on the maze level). Each rat was gently placed in the center of the plus-maze, facing one of the closed arms, immediately after a 5-min adaptation period in a plastic black box (60 × 60 × 35 cm), to increase the overall activity in the EPM. During a 5-min test period, automated Motor Monitor System recorded the number of entries into the closed and open arms and the time spent in either type of arms. The device counted an effective arm-entry when the four paws of a rat were into any arm. The maze was thoroughly cleaned after each trial. The EPM test is an “unconditional” anxiety-like test based on rodents’ natural aversion to heights and open space.

##### Exploratory Activity Measured in the EPM Test

The experiment was performed using an EPM apparatus (details see above). Total ambulation (the total distance covered by a rat, and ambulation along X and Y axis) was taken to discern drug effects on general activity from those on open-arm exploration, during a 5-min test period (i.e., the time equal to the observation period in the EPM test). Rats’ behavior was not videotaped during the test.

### 4.7. Statistical Analysis

The GraphPad Prism™ 6 software was used to calculate statistical significances in ADMET and in vivo experiments. 

The statistical significances were evaluated by an analysis of variance one-way ANOVA followed by Bonferroni’s post-hoc test (statistical significance set at *p* < 0.05) in ADMET, FST, EPM and NOR tests. Results of behavioral studies are given as mean ± standard error of the mean (SEM).

Differences in pharmacokinetic parameters between the compounds **2** and **3** were evaluated using Student’s *t*-test and Mann–Whitney U-test where appropriate after normality was tested using the Shapiro–Wilk test. Pharmacokinetic parameters were expressed as the mean ± SD. Statistical significance was defined as *p* < 0.05. All calculations were carried out by the TIBCO Statistica 13.3 software (Software Inc. Palo Alto, CA, USA). 

## 5. Conclusions

Within this study, three 5-HT_6_R triazine agents, i.e., the 2,5-dichlorophenyl- (**1**), the 2,3-dichlorophenyl (**2**) and the unsubstituted phenyl (**3**) ones, were investigated. The receptor profile in radioligand binding and functional assays, ADMET properties, including PAMPA permeability, Caco-2 permeability assay, the microsome metabolic stability as well as DDI, hepatotoxic, mutagenic and neurotoxic effects were estimated in vitro, while pharmacokinetics and procognitive-like, antidepressant-like and anxiolytic-like properties of the triazine-compounds were examined in vivo. 

A comprehensive analysis of the obtained results indicated significant procognitive potential together with beneficial ADMET in vitro and pharmacokinetics in vivo profiles for both, (*RS*)-4-[1-(2,3-dichlorophenoxy)propyl]-6-(4-methylpiperazin-1-yl)-1,3,5-triazin-2-amine (**2**) and (*RS*)-4-(4-methylpiperazin-1-yl)-6-(1-phenoxypropyl)-1,3,5-triazin-2-amine (**3**), but insensibly predominant for compound **2**. Interestingly, both compounds (**2** and **3**) also exhibit protective activity against rotenone-induced neurotoxicity in SH-SY5Y neuroblastoma cells. Thus, it can be presumed that they seem to be good CNS-drug candidates useful for further development in search for new drugs against dementia disorders, including Alzheimer’s disease.

## Figures and Tables

**Table 1:** The affinities for serotonin/dopamine receptors of the compounds **1–3**.

Cpd	*K_i_* (nM) ± SD				
	^a^ 5-HT_6_	^a^ D_2_	5-HT_1A_	^a^ 5-HT_2A_	^a^ 5-HT_7_
**1** **2** **3**	6 ± 1 6 ± 2 21 ± 5	320 ± 19 421 ± 29 1506 ± 338	4760 ± 1191 521 ± 73 3643 ± 816	484 ± 77 209 ± 19 5047 ± 752	5706 ± 967 5202 ± 1247 19,940 ± 2357
Ref ^b^	7 ± 1	9 ± 2	-	-	-

SD—standard deviation; ^a^ receptors affinity that was revealed previously [21]; ^b^ olanzapine.

**Table 2:** The results from functional assays towards 5-HT_6_R and 5-HT_2A_R for compounds **1–3**.

Target	5-HT_2A_R		5-HT_6_R	
Compound	Agonist Mode * E_max_ [%] ± SEM	Antagonist Mode ** *pK_b_* ± SEM	Agonist Mode * E_max_ [%] ± SEM	Antagonist Mode *** *pK_b_* ± SEM
Serotonin	100.00 ± 0.30	N.C.	100.00 ± 0.5	N.C.
Mianserin	1.00 ± 0.30	9.70 ± 0.12	2.00 ± 0.50	6.08 ± 0.05
SB258585	N.T.	N.T.	2.00 ± 0.50	8.92 ± 0.10
**1**	58.00 ± 9.70	N.C.	4.00 ± 0.00	10.57 ± 0.07
**2**	21.00 ± 0.60	8.61 ± 0.03	5.00 ± 1.10	8.19 ± 0.33
**3**	N.T.	N.T.	9.00 ± 1.40	7.67 ± 0.20

* Results were normalized as percentage of maximal agonist response (Serotonin 10^−5^ M). ** Results were normalized as percentage of maximal response in the absence of antagonist. *** Results were normalized as percentage of reference antagonist (SB258585 10^−5^ M). E_max_ is the maximum possible effect. N.C.—not calculable. N.T.—not tested.

**Table 3:** The results obtained in PAMPA for 5-HT_6_R ligands and the reference caffeine (CFN).

Cpd	^*a*,*b*^*Pe* (10^−6^ cm/s) ± SD
CFN	15.1 ± 0.4
**1**	18.9 ± 0.9 ^c^
**2**	20.5 ± 5.4
**3**	15.1 ± 1.0

***^a^*** PAMPA plate’s manufacturer breakpoint for permeable compounds: *Pe* ≥ 1.5 × 10^−^^6^ cm/s [22]; ***^b^*** tested in triplicate; ***^c^*** *Pe* value revealed in previous studies [21].

**Table 4:** The results obtained in a bi-directional Caco-2 permeability assay for 5-HT_6_R ligands and the reference.

Cpd	^a^*Papp* (10^−6^ cm/s) ± SD		^b^ Efflux Ratio
	A-B	B-A	
CFN	15.6 ± 0.55	17.9 ± 1.9	1.14
**1**	14.2 ± 0.36	22.2 ± 1.92	1.56
**2**	50.3 ± 6.22	21.1 ± 2.14	0.42
**3**	3.16 ± 1.35	10.1 ± 0.15	3.20

^a^ *Papp*, Apparent permeability coefficient, test permeability for each compound was tested in triplicate, data are mean ± standard deviation. ^b^ The quotient of mean *Papp* for B-A to the mean *Papp* for A-B.

**Table 5:** The molecular masses and metabolic pathways of compounds **2** and **3**.

Substrate	Molecular Mass (*m*/*z*)	Amount of Metabolites	Molecular Mass of the Metabolite (*m*/*z*)	Content in Reaction Mixture (%)	Metabolic Pathway *
**2**	397.31	3	413.07 (**M1**) 413.07 (**M2**) 383.03 (**M3**)	13.53 2.57 1.70	*hydroxylation* *hydroxylation* *demethylation*
**3**	328.42	2	279.22 (**M1**) 345.28 (**M2**)	2.56 1.77	*decomposition and triple hydroxylation* *hydroxylation*

* Estimated according to MS spectra supporting by in silico data (see Supplementary 3.0).

**Table 6:** The mutagenicity potential of **1–3**, reference mutagen nonyl-4-hydroxyquinoline-*N*-oxide (NQNO, 0.5 µM).

Conc. (µM)	Compound	Fold Increase over MCB ^a^	Binomial B-Value ^b^	Number of Revertants ± SD
0.5	NQNO	3.23	1.0000	48.00 ± 0.00
1	**2**	0.81	0.2601	12.00 ± 7.55
10	**2**	0.90	0.5425	13.33 ± 2.31
0.25	NQNO	7.02	1.0000	40.33 ± 4.04
1	**1**	0.35	0.0707	2.00 ± 1.00
10	**1**	0.35	0.0707	2.00 ± 1.00
1	**3**	0.06	0.0001	0.33 ± 0.58
10	**3**	0.29	0.0324	1.67 ± 1.53

^a^ Fold increase over MCB (baseline)values ≥ 2.0 scores individual doses as positive. MCB (baseline) means positive wells+1SD in the control. ^b^ Binomial B-value indicates the probability that spontaneous mutation events alone. The result ≥0.99 indicates that chances of spontaneous mutation are ≤1%.

**Table 7:** Pharmacokinetic parameters calculated non-compartmental analysis from the concentration of **2** and **3** compounds in serum after single i.p. administration at the doses of 0.3 mg/kg (mean ± SD, *n* = 4/group).

Pharmacokinetic Parameter ^1^	2	3
C_max_ (ng/mL)	5.20 ± 2.24	6.32 ± 5.03
T_max_ (min)	15	5
AUC_0-t_ (ng∙min/mL)	453.40 ± 145.83	423.91 ± 216.75
AUC_0-∞_ (ng∙min/mL)	530.79 ± 150.52	511.88 ± 262.32
V_z_/F (L/kg)	80.99 ^a^ ± 18.22	64.81 ± 21.78
CL/F (L/h/kg)	30.87 ± 9.88	35.16 ± 17.92
t_1/2λz_ (min)	116.18 ^a^ ± 17.14	76.65 ± 18.07
MRT (min)	90.08 ± 7.02	79.33 ± 6.56

^1^ C_max_—maximum concentration; T_max_—time to reach the maximum concentration; AUC_0-∞_—area under the plasma concentration–time curve from time zero to infinity; V_z_/F—volume of distribution at the elimination phase; CL/F—oral clearance; t_1/2λz_—half-life in the elimination phase; MRT—mean residence time; ^a^ *p* < 0.05 between pharmacokinetic parameters.

**Table 8:** Mean values of pharmacokinetic parameters calculated from the concentrations of **2** and **3** compounds in serum and tissues after single i.p. administration at a dose of 0.3 mg/kg (non-compartmental analysis) (*n* = 4/time point).

2				
Tissue	T_max_ (min)	C_max_ (ng/mL or ng/g)	AUC_0-t_ (ng·min/mL or ng·min/g)	K_p_
Serum	15	5.20 ± 2.24	453.40 ± 145.83	-
Brain	5	11.63 ± 3.13	1219.94 ± 368.79	2.69
Heart	5	8.90 ± 8.15	1040.74 ± 448.65	2.30
Lungs	5	54.20 ± 21.61	3870.66 ± 680.07	8.54
Liver *	15	115.22 ± 93.46	6523.60 ± 4997.28	14.39
Kidneys	15	65.73 ± 34.94	7488.06 ± 4372.92	16.52
**3**				
Serum	5	6.32 ± 5.04	423.91 ± 216.75	-
Brain	5	11.35 ± 6.93	859.08 ± 312.87	2.03
Heart	5	21.83 ± 17.16	1057.37 ± 285.41	2.49
Lungs	5	73.65 ± 63.94	2715.09 ± 1000.42	6.41
Liver *	5	129.62 ± 106.23 **	4467.18 ± 2249.99	10.54
Kidneys	15	41.25 ± 8.41	2557.08 ± 1084.42	6.03

C_max_—maximum concentration; T_max_—time to reach the maximum concentration; K_p_—ratio of AUC_0-t_ tissue to AUC_0-t_ serum. * C_max_ ratio (2/3) = 0.89. ** Expressed with molar concentration: C_max_ = 0.395 µM.

**Table 9:** The effect of compounds **2** and **3** on the exploration activity of rats in the NOR test ^1^.

Treatment	Dose (mg/kg)	Total Exploratory Time in T2 Session (s)
Vehicle + vehicle	0 + 0	58.71 ± 3.38
MK-801 + vehicle	0.1 + 0	42.00 ± 3.13
**2** + MK-801	0.3 + 0.1	52.67 ± 5.37
	1 + 0.1	40.33 ± 6.66
	3 + 0.1	42.88 ± 2.10 F(4,27) = 3.5450; *p* < 0.05
vehicle	0 + 0	45.00 ± 4.30
MK-801 + vehicle	0.1 + 0	47.07 ± 3.89
**3** + MK-801	0.1 + 0.1	47.33 ± 4.79
	0.3 + 0.1	38.14 ± 4.18
	1 + 0.1	35.57 ± 3.76
	3 + 0.1	34.14 ± 1.22; F(5,47) = 1.9394; NS

^1^ Compounds **2** and **3** were given i.p. 60 min, while MK-801 was injected i.p. 30 min before the test. Values represent the mean ± SEM of the total exploratory time of both objects during the 3-min test session (T2) compared to the respective vehicle group (one-way ANOVA followed by Bonferroni’s post-hoc test); NS—non-significant. N = 6–7.

**Table 10:** Effect of compounds **1**, **2**, **3** on total exploration in the EPM test in rats ^1^.

Treatment	Dose (mg/kg)	Total Distance (cm)	X Ambulation	Y Ambulation
Vehicle	0	4600 ± 140	175 ± 7	104 ± 10
1	1	4520 ± 168	172 ± 6	99 ± 6
	3	3995 ± 218	146 ± 10	82 ± 7
	10	3681 ± 256; *p* < 0.05 F(3,24) = 4.5291; *p* < 0.05	140 ± 11 F(3,24) = 3.8035; *p* < 0.05	83 ± 8 F(3,24) = 1.9215; NS
Vehicle	0	3639 ± 327	137 ± 19	81 ± 12
2	0.3	4372 ± 132	180 ± 9	101 ± 9
	1	3987 ± 281	149 ± 18	78 ± 9
	3	4595± 191 F(3,23) = 2.9772; NS	168 ± 6 F(3,23) = 1.9417; NS	115 ± 7 F(3,23) = 3.1727; NS
Vehicle	0	3639 ± 327	137 ± 19	81 ± 12
3	0.3	4033 ± 340	151 ± 16	80 ± 8
	1	4102 ± 291	136 ± 15	86 ± 10
	3	3545 ± 136 F(3,22) = 0.9071; NS	123 ± 5 F(3,22) = 0.6336; NS	73 ± 5 F(3,22) = 0.3805; NS

^1^ Compounds **1**–**3** were given i.p. 60 min before the test. Values represent the mean ± SEM of the total distance, X ambulation, and Y ambulation during 5-min test session compared to the respective vehicle group (one-way ANOVA followed by Bonferroni’s post hoc test); NS—non-significant. *N* = 6–7.

## Supplementary Materials

The following are available online at https://www.mdpi.com/article/10.3390/ijms221910773/s1, Figure S1: (**a**) MS spectra of compound **2**, control. (**b**) MS spectra and the most probable structure of compound’s **2** metabolite M1 obtained after 120 min incubation with RLMs. (**c**) MS spectra and the most probable structure of compound’s **2** metabolite M2 obtained after 120 min incubation with RLMs. (**d**) MS spectra and the most probable structure of compound’s **2** metabolite M3 obtained after 120 min incubation with RLMs, Figure S2: (**a**) MS spectra of compound **3**, control. (**b**) MS spectra and the most probable structure of compound’s **3** metabolite M1 obtained after 120 min incubation with RLMs. (**c**) MS spectra and the most probable structure of compound’s **9** metabolite M2 obtained after 120 min incubation with RLMs, Table S1: Results of functional assay towards 5-HT6, Table S2: Results of functional assay towards 5-HT2A, Table S3: The metabolic pathways of compounds **2** and **3**, Table S4: The impact of compounds **1–3** on the MK-801-induced memory impairment in the NOR test, Table S5: Effect of compounds **1**, **2**, **3** on the immobility time in FST in rats, Table S6: Effects of compounds **1**, **2**, **3** in the EPM test in rats.
