# Supplementary material for: The Phenoxyalkyltriazine Antagonists for 5-HT6 Receptor with Promising Procognitive and Pharmacokinetic Properties In Vivo in Search for a Novel Therapeutic Approach to Dementia Diseases
Source: Int J Mol Sci. 2021 Oct 5;22(19):10773. doi: 10.3390/ijms221910773 (PMC8509428; doi:10.3390/ijms221910773)
Supplement: Supplementary file 1 [file ijms-22-10773-s001.zip › ijms-1342973-supplementary.pdf]

International Journal of *Molecular Sciences*

MDPI

Supplemnetary materials

Table S1. Results of functional assay towards 5-HT_6.

	Agonist Mode*					Antagonist Mode**											
	E max (%)	SEM	EC50	pEC50	SEM		E max (%)	SEM	IC50	IC50	pIC50	SEM	Kb	Kb	pKb	SEM	R2
			M		pEC 50				M	nM			M	nM		pKb	Kb
Serotonin	100	0.5	2.76 × 10^{-9}	8.6	0	Sero-tonin	0.0	1.0	N.C	N.C	N.C	N.C	N.C	N.C	N.C	N.C	N.C
SB 258585	2	0.5	N.C.	N.C.	N.C.	SB 258585	100	0.7	6.01 × 10^{-9}	6.0	8.22	0,10	1.20 × 10^{-9}	1.2	8.92	0.10	0.945
Mianseri	2	0.5	N.C.	N.C.	N.C.	MI-AN-SER-IN	91	4.0	4.15 × 10^{-6}	4152.0	5.38	0,05	8.31 × 10^{-7}	830.5	6.08	0.05	0.988
**1**	4	0.5	N.C.	N.C.	N.C.	**1**	98	1.3	1.40 × 10^{-10}	1400.0	9.87	0.065	2.70 × 10^{-11}	0.027	10.57	0.0065	0.907
**2**	5	1.1	N.C.	N.C.	N.C.	**2**	96	3.1	6.33 × 10^{-8}	63.3	7.12	0,33	6.46 × 10^{-9}	6.5	8.19	0.33	0.903
**3**	9	3.3	N.C.	N.C.	N.C.	**3**	100	0.7	1.07 × 10^{-7}	106.6	6.97	0,20	2.13 × 10^{-8}	21.3	7.67	0.20	0.972

*Results were normalized as percentage of maximal agonist response (Serotonin 10^{-5} M) **Results were normalized as percentage of reference antagonist (SB258585 10^{-5} M) Emax is the maximum possible effect.

N.C.—not calculable.

Table S2. Results of functional assay towards 5-HT_{2A}.

	Agonist Mode*				Antagonist mode**									
	E_{max} (%)	SEM	EC_{50}	pEC_{50}		E_{max} (%)	SEM	IC_{50}	IC_{50}	pIC_{50}	K_b	K_b	pK_b	R^2
			M					M	nM		M	nM		
Serotonin	100	0.3	1.45 E-09	8.84	Sero-tonin	1	0.3	N.C.	N.C.	N.C.	N.C.	N.C.	N.C.	N.C.
SB 258585	N.T.													
Mianserin	1	0.3	N.C.	N.C.	Mi-an-serin	1	0.3	1.01 × 10^{-9}	1.01	9.00	2.01 × 10^{-10}	0.20	9.70	0.928

**1**	58	9.7	4.49 × 10^{-8}	7.35	**1**	0	0	N.C.	N.C.	N.C.	N.C.	N.C.	N.C.	N.C.
**2**	21	0.6	5.84 × 10^{-7}	6.22	**2**	1	0.3	1.24 × 10^{-8}	12.36	7.90	2.47 × 10^{-9}	2.47	8.61	0.984
**3**	N.T.													

*Results were normalized as percentage of maximal agonist response (Serotonin 10^{-5} M) **Results were normalized as percentage of reference antagonist (SB258585 10^{-5} M) Emax is the maximum possible effect.
N.C.—not calculable N.T.—not tested.

Table S3. The metabolic pathways of compounds **2** and **3**.

Substrate	Molecular Mass (m/z)	Retention Time (min.)	Molecular Mass of the Metabolite (m/z)	Metabolic Pathway	Probable Structures of Metabolites*
**2**	397.31		413.07 (**M1**)<br>413.07 (**M2**)<br>383.03 (**M3**)	hydroxylation<br>hydroxylation<br>demethylation	Fig. S1B<br>Fig. S1C<br>Fig. S1D
**3**	328.42		279.22 (**M1**)<br><br>345.28 (**M2**)	decomposition and triple hydroxylation<br>hydroxylation	Fig. S2B<br><br>Fig. S2C

Table S4. The impact of compounds **1–3** on the MK-801-induced memory impairment in the NOR test.

Treatment	Dose (mg/kg)	Discrimination Index
Vehicle	0 + 0	0.37 ± 0.03
MK-801 + Vehicle [17]	0.1+0	−0.07 ± 0.01; $p < 0.05$ vs veh
**1** + MK-801 [17]	0.3 + 0.1	0.29 ± 0.04; ns vs veh,; ns vs MK
	1+0.1	0.13 ± 0.08; ns vs veh; ns vs MK
	3+0.1	0.42 ± 0.18; ns vs veh; $p < 0.05$ vs MK<br>$F(4,30) = 3.9851$; $p < 0.01$
Vehicle + Vehicle	0 + 0	0.21 ± 0.05
MK-801 + Vehicle	0.1+0	−0.05 ± 0.03; $p < 0.01$ vs veh
**2** + MK-801	0.3 + 0.1	0.26 ± 0.04; ns vs veh; $p < 0.01$ vs MK
	1+0.1	0.19 ± 0.04; ns vs veh; $p < 0.05$ vs MK
	3+0.1	0.30 ± 0.06; ns ve veh, $p < 0.001$ vs MK<br>$F(4,27) = 7.5715$; $p < 0.001$

Vehicle	0 + 0	0.27 ± 0.04
MK-801 + vehicle	0.1+0	-0.11 ± 0.07; $p < 0.05$ vs veh
**3** + MK-801	0.1 + 0.1	0.26 ± 0.04; ns vs veh; $p < 0.01$ vs MK
	0.3 + 0.1	0.30 ± 0.04; ns vs veh, $p < 0.001$ vs MK
	1+0.1	0.18 ± 0.15; ns vs veh, $p < 0.05$ vs MK
	3+0.1	0.31 ± 0.05, ns vs veh, $p < 0.001$ vs MK<br>F(5,47) = 8.1658; $p < 0.0001$

Compounds **1**, **2**, **3** were given *i.p.* 60 min while MK-801 was given *i.p.* 30 min before the T1 session. Values represent the mean ± SEM of the discrimination index during 3-min test session compared to the respective vehicle group (one-way ANOVA followed by Bonferroni's post-hoc test); NS—non-significant. N=6-7.

Table S5. Effect of compounds **1**, **2**, **3** on the immobility time in FST in rats.

Treatment	Dose (mg/kg)	Immobility Time (s)
Vehicle	0	259.86 ± 5.4
**1**	1	241.80 ± 18.7
	3	175.29 ± 18.1; $p < 0.01$
	10	163.63 ± 11.2; $p < 0.001$<br>F(3,26) = 10.615; $p < 0.001$
Vehicle	0	227.71 ± 12.7
**2**	1	223.13 ± 11.4
	3	220.71 ± 4.7
	10	171.38 ± 12.80; $p < 0.01$<br>F(3,26) = 5.8561; $p < 0.01$
Vehicle	0	211.00 ± 9.3
**3**	1	177.50 ± 6.1
	3	159.13 ± 13.3; $p < 0.05$
	10	134.63 ± 16.7; $p < 0.001$<br>F(3,26) = 6.8104; $p < 0.01$

Decreased immobility time denotes antidepressant-like activity. Compounds **1**, **2**, **3** were given *i.p.* 60 min before the test. Values represent the mean ± SEM of immobility time during 5-min test session compared to the respective vehicle group (one-way ANOVA followed by Bonferroni's post-hoc test); *N*=6-8.

Table S6. Effects of compounds **1**, **2**, **3** in the EPM test in rats.

Treatment	Dose	Open Arms				
	(mg/kg)	Time (s)	% of time	Entries	% of entries	Distance (cm)
Vehicle	0	31.39 ± 10.11	15.00 ± 4.29	9.14 ± 1.98	29.44 ± 4.82	456 ± 137
**1**	1	58.90 ± 14.20	25.76 ± 6.11	9.88 ± 1.99	32.59 ± 4.23	788 ± 175
	3	24.81 ± 5.24	10.27 ± 2.05	6.14 ± 0.77	29.49 ± 3.48	307 ± 78
	10	43.36 ± 9.08<br>F(3,23) = 2.1368;	18.34 ± 4.19<br>F(3,23) = 2.3504; NS	7.20 ± 1.66<br>F(3,23) = 1.0379; NS	42.23 ± 9.09<br>F(3,23) = 1.2608; NS	538 ± 104<br>F(3,23) = 2.3440; NS

		NS				
Vehicle	0	32.63 ± 9.50	12.67 ± 3.40	5.00 ± 1.20	24.90 ± 1.90	390 ± 116
**2**	0.3	36.85 ± 8.90	14.85 ± 3.30	6.50 ± 1.60	23.97 ± 4.97	423 ± 87
	1	24.75 ± 3.19	10.10 ± 1.20	5.86 ± 0.90	26.53 ± 3.69	279 ± 45
	3	72.23 ± 7.30; $p < 0.05$ F(3,23) = 6.9314; $p < 0.01$	30.00 ± 3.60; $p < 0.01$ F(3,23) = 7.7627; $p < 0.001$	13.00 ± 2.30; p<0.05 F(3,23) = 5.0274; $p < 0.01$	41.89 ± 3.37 F(3,23) = 3.9489; NS	914 ± 126; $p < 0.01$ F(3,23) = 8.2188; $p < 0.001$
**Vehicle**	0	32.63 ± 9.50	12.67 ± 3.50	5.00 ± 1.20	24.90 ± 1.90	390 ± 116
**3**	0.3	24.27 ± 3.41	9.84 ± 1.50	5.28 ± 0.42	28.54 ± 6.70	340 ± 59
	1	44.24 ± 9.71	18.30 ± 4.10	8.43 ± 1.36	33.64 ± 2.70	646 ± 145
	3	19.00 ± 5.50 F(3,22) = 2.1964; NS	7.03 ± 2.00 F(3,22) = 2.6159; NS	4.17 ± 1.10 F(3,22) = 3.0956; NS	24.39 ± 7.60 F(3,22) = 0.6565; NS	231 ± 87 F(3,22) = 2.7192; NS

Increased open-arm exploration denotes reduced anxiety. Compounds **1**, **2**, **3** were given *i.p.* 60 min before the test. Values represent the mean ± SEM of the time and percentage of time spent in open arms, entries and percentage of entries into the open arms during 5-min test session compared to the respective vehicle group (one-way ANOVA followed by Bonferroni's post-hoc test); NS—non-significant. N=6-7.

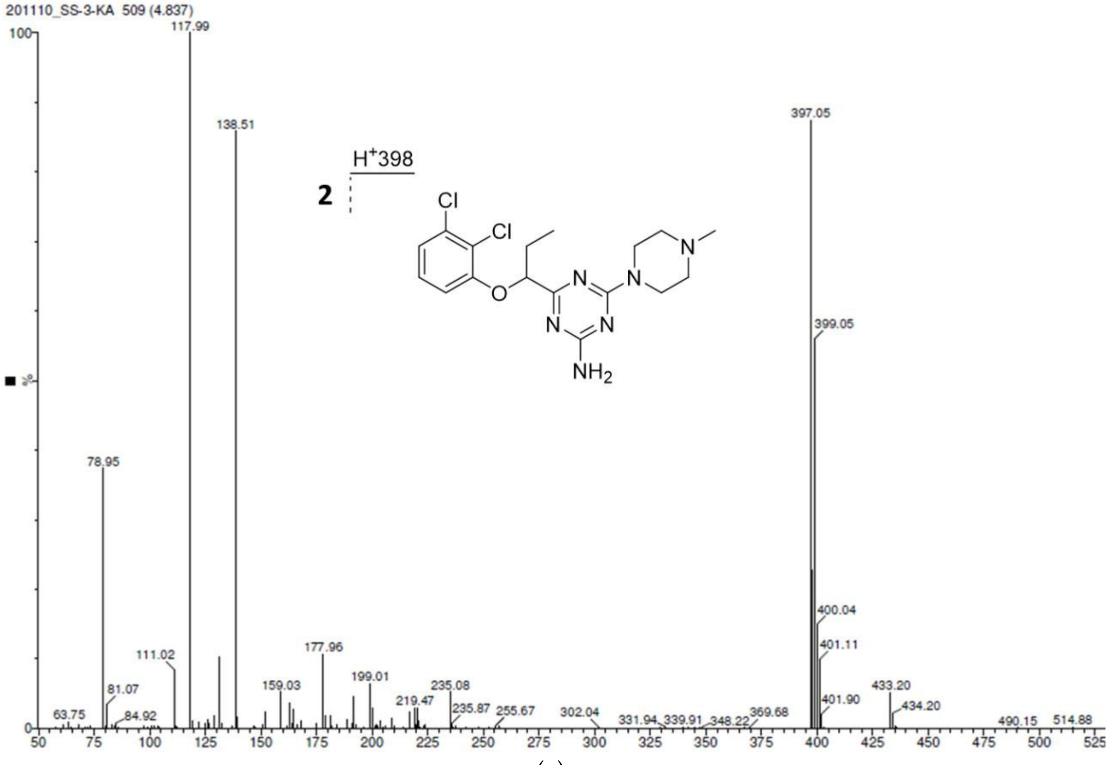

(**a**)

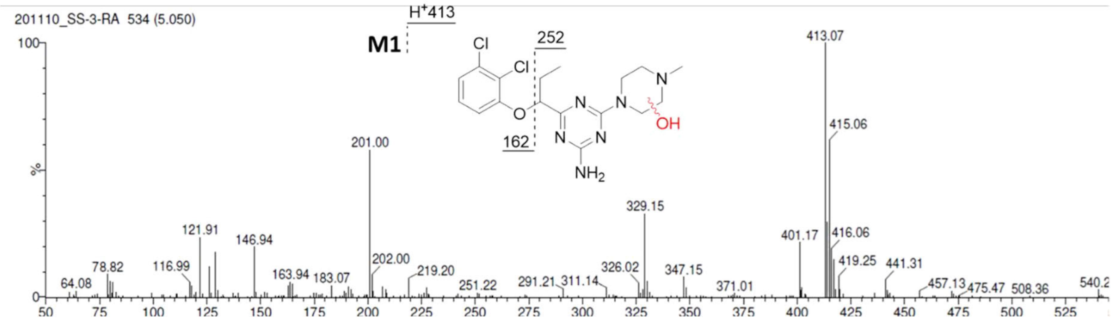

(**b**)

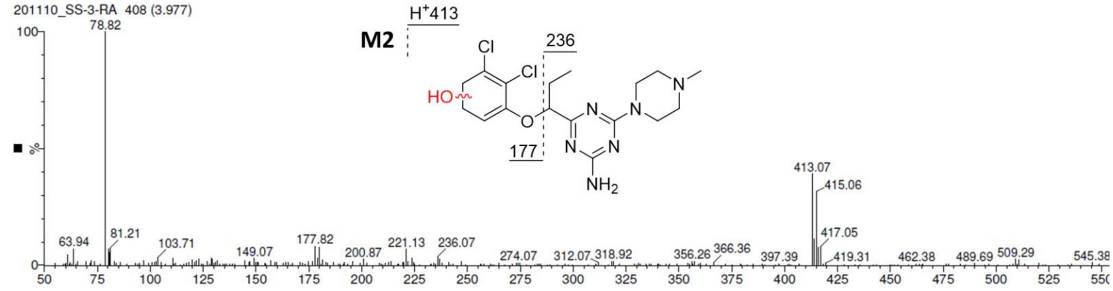

(**c**)

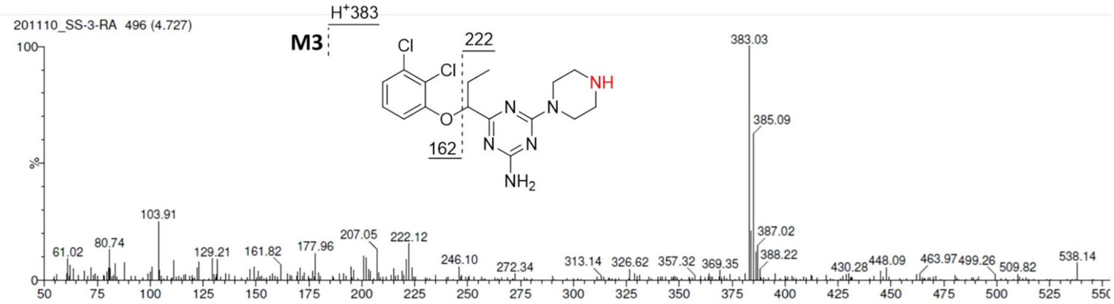

(**d**)

Figure S1. (**a**) MS spectra of compound **2**, control. (**b**) MS spectra and the most probable structure of compound's **2** metabolite M1 obtained after 120 min incubation with RLMs. (**c**) MS spectra and the most probable structure of compound's **2** metabolite M2 obtained after 120 min incubation with RLMs. (**d**) MS spectra and the most probable structure of compound's **2** metabolite M3 obtained after 120 min incubation with RLMs.

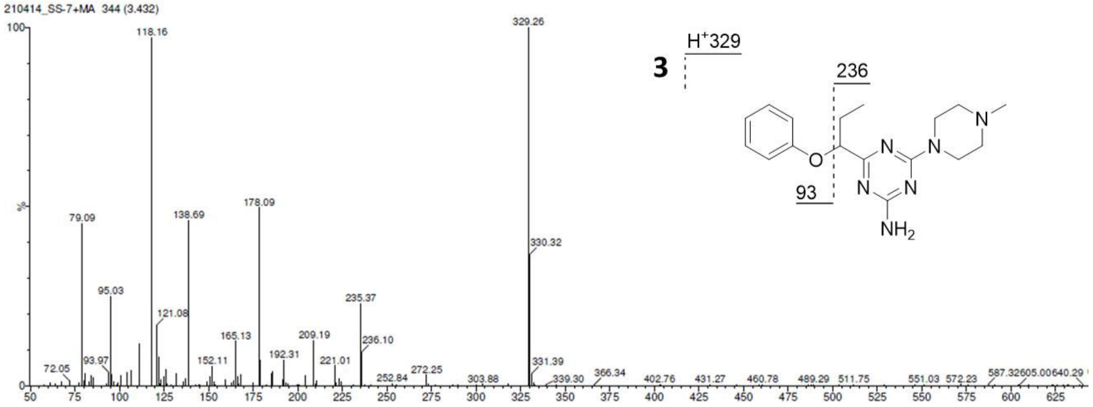

(**a**)

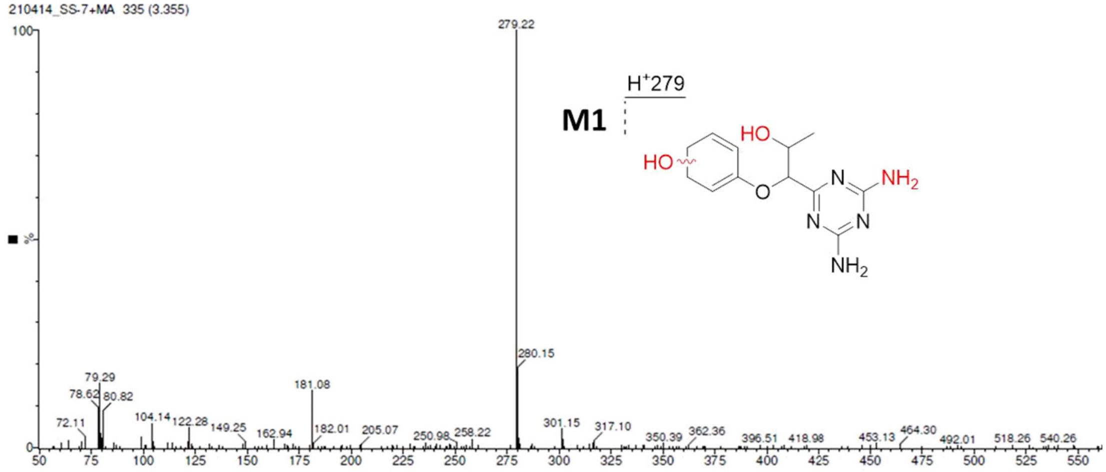

(**b**)

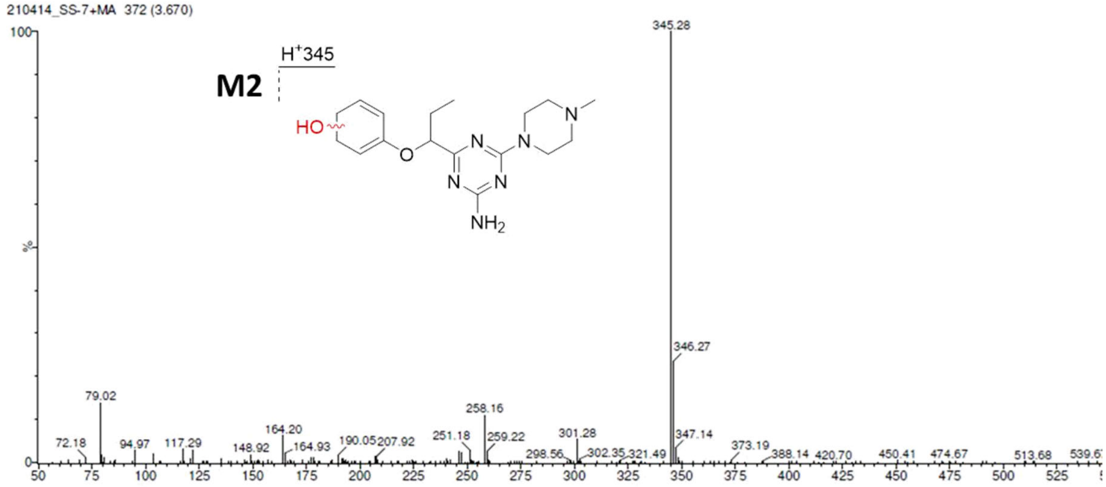

(**c**)

Figure S2. (**a**) MS spectra of compound **3**, control. (**b**) MS spectra and the most probable structure of compound's **3** metabolite M1 obtained after 120 min incubation with RLMs. (**c**) MS spectra and the most probable structure of compound's **9** metabolite M2 obtained after 120 min incubation with RLMs.
